# Evidence That Rhesus Macaques Self-Cure from a *Schistosoma japonicum* Infection by Disrupting Worm Esophageal Function: A New Route to an Effective Vaccine?

**DOI:** 10.1371/journal.pntd.0003925

**Published:** 2015-07-10

**Authors:** Xiao-Hong Li, Yu-Xin Xu, Gill Vance, Yun Wang, Long-Bao Lv, Govert J. van Dam, Jian-Ping Cao, R. Alan Wilson

**Affiliations:** 1 National Institute of Parasitic Diseases, Chinese Center for Disease Control and Prevention, Shanghai, China; 2 Key Laboratory of Parasitology and Vector Biology, Ministry of Health, Shanghai, China; 3 Centre for Immunology and Infection, Department of Biology, University of York, York, United Kingdom; 4 Kunming Institute of Zoology, Chinese Academy of Science, Kunming, China; 5 Department of Parasitology, Leiden University Medical Centre, Leiden, The Netherlands; McGill University, CANADA

## Abstract

**Background:**

Rhesus macaques are unusual among schistosome hosts, self-curing from an established infection and thereafter manifesting solid immunity against a challenge, an ideal model for vaccine development. Previously, the immunological basis of self-cure was confirmed; surviving worms had ceased feeding but how immunological pressure achieved this was unclear. The schistosome esophagus is not simply a conduit for blood but plays a central role in its processing. Secretions from the anterior and posterior esophageal glands mix with incoming blood causing erythrocyte lysis and tethering and killing of leucocytes.

**Methodology/Principal Findings:**

We have analysed the self-cure process in rhesus macaques infected with *Schistosoma japonicum*. Faecal egg output and circulating antigen levels were used to chart the establishment of a mature worm population and its subsequent demise. The physiological stress of surviving females at perfusion was especially evident from their pale, shrunken appearance, while changes in the structure and function of the esophagus were observed in both sexes. In the anterior region electron microscopy revealed that the vesicle secretory process was disrupted, the tips of lining corrugations being swollen by greatly enlarged vesicles and the putative sites of vesicle release obscured by intense deposits of IgG. The lumen of the posterior esophagus in starving worms was occluded by cellular debris and the lining cytoplasmic plates were closely adherent, also potentially preventing secretion. Seven proteins secreted by the posterior gland were identified and IgG responses were detected to some or all of them. Intrinsic rhesus IgG colocalized with secreted SjMEGs 4.1, 8.2, 9, 11 and VAL-7 on cryosections, suggesting they are potential targets for disruption of function.

**Conclusions/Significance:**

Our data suggest that rhesus macaques self-cure by blocking esophagus function with antibody; the protein products of the glands provide a new class of potential vaccine targets.

## Author Summary

Rhesus macaques can self-cure from a schistosome infection. Antibody is crucial to drive this process and adult worm elimination is preceded by cessation of blood feeding. Recently we have shown that the schistosome esophagus plays a central role in blood processing. We first confirm the self-cure process in rhesus macaques infected with *Schistosoma japonicum* and provide evidence that the self-cure mechanism involves blocking the worm esophagus function with antibody. In the anterior region, secretion of light vesicles is disrupted hence their contents are not released into the lumen to interact with blood components to fulfil their tasks. The plates in the posterior lining stick together whilst the lumen is occluded, hampering blood processing. Furthermore, rhesus IgG binds strongly to the worm esophageal lumen and co-localizes completely with five esophageal secreted proteins, SjMEGs 4.1, 8.2, 9, 11 and VAL-7. Our results indicate that rhesus macaques eliminate their adult worms by disrupting esophageal function making blood difficult to ingest; feeding stops eventually causing their demise because nutrient uptake across the body surface cannot fully compensate.

## Introduction

Schistosomiasis is one of the most important parasitic diseases in tropical and sub-tropical regions of the world, with about 800 million people at risk and more than 200 million infected [1,2]. With intense efforts over six decades, great progress has been made in combating this disease in China [3], nevertheless zoonotic schistosomiasis japonica remains a major public health problem. Although comprehensive measures, including mass treatment, snail control and environmental modifications have proved effective in reducing the prevalence and morbidity in endemic areas, none of them can prevent re-infection. Furthermore, unlike other schistosome species, *Schistosoma japonicum* has a wide range of reservoir hosts in China [4]; 42 species in 28 genera within seven orders of mammals can harbor the infection naturally, adding considerably to the complexity of disease control and prevention. A vaccine with long-term efficacy would augment efforts to control and ultimately eradicate the disease and hence has received wide attention but so far has proved to be an elusive goal.

After rhesus macaques are exposed to cercariae of *S*. *japonicum*, an infection becomes patent and the animals manifest all the characteristics of a permissive host. However, unusually among all the natural and laboratory hosts, after a lag period of up to 12 weeks, faecal egg output drops sharply and eventually declines to zero [5,6]. The females recovered after this time were reduced in size with smaller ovaries and fewer eggs in the uterus [7]. These observations on the fate of a schistosome infection in rhesus macaques are common to both Japanese and Chinese mainland strains of *S*. *japonicum* [6,7] as well as two other human species, *S*. *mansoni* [8,9] and *S*. *haematobium* [10]. That they might represent a ‘self-cure’ from the schistosome infection was first proposed by Cheever et al. [6], although a clear definition was not articulated. Here we contend that a phenomenon where a patent infection establishes but worms are subsequently eliminated without any external intervention, has indeed all the hallmarks of a self-cure process.

Although a number of studies on rhesus monkeys have demonstrated complete or partial self-cure, they provide few clues about the nature of the mechanism that drives the process. One reason is that most were carried out between the 1940s and 1970s when knowledge of immune effector mechanisms was quite limited. A frequent observation was that successful self-cure was related to initial cercarial dose, with only higher intensity infections bringing about almost complete worm elimination [9]. Most importantly, animals that self-cured showed a strong resistance to a challenge infection with normal cercariae [10,11,12]. Such resistance was only complete when a challenge exposure was applied to animals whose self-cure was well underway [10,13], indicating that resistance developed slowly. However, once protective immunity was established, it did not require any surviving adult worms for its maintenance because when they were killed by drug treatment, it still persisted [13]. More convincing evidence for an immunological basis to self-cure finally came decades later [14], where the timing and intensity of IgG production was shown to correlate inversely with the number of *S*. *mansoni* worms recovered by perfusion at 18 weeks. No evidence was found for an acute antibody-mediated lethal hit. Instead the mechanism appeared to involve cessation of blood feeding, starvation and ultimately organ failure. These conclusions were reinforced by the retarded growth of blood-feeding worms during in vitro culture with rapid-responder serum versus slow-responder or naive rhesus serum.

Schistosomes feed avidly on blood, with male and female adult *S*. *mansoni* ingesting some 39,000 and 330,000 erythrocytes per hour, equating to daily intakes of 105 nl and 880 nl of whole blood, respectively [15]. We recently demonstrated that the worm esophagus does not act simply as a conduit but plays a central role in blood processing [16,17]. Erythrocytes are lysed there whilst leucocytes are tethered and damaged, forming a stationary plug in the posterior esophageal lumen around which blood flows. The posterior region is surrounded by a gland and, in *S*. *japonicum* at least, the anterior esophageal region is also a morphologically distinct secretory organ [18]. Very little is known about the products of these two glands, in part due to their minute size. Expression of four genes, Micro Exon Genes (MEGs) 4.1, 4.2, 14 and Venom Allergen Like (VAL)-7 [17,19,20] exclusively in the posterior gland of *S*. *mansoni* was revealed by whole mount in-situ hybridization (WISH). MEG-4.1 protein expression was also demonstrated in the posterior gland of *S*. *japonicum* [17]. So far only one single gene MEG-12, has been identified from this newly discovered anterior esophageal gland, whose protein product was shown to destabilize erythrocyte membranes (R DeMarco, personal communication).

Here we have made an in-depth exploration of the self-cure process in rhesus macaques infected with *S*. *japonicum* (China mainland strain). We examined the morphological and physiological aspects of the parasites, as well as host responses to them. We have also explored the possible mechanisms of self-cure and potential immune targets of the rhesus macaque host, against the background of new findings on schistosome feeding [21]. An ideal model on which vaccine development can be based should satisfy the dual criteria that high protection (up to 100%) is induced and persistent immunity is maintained. In this context, we believe our findings with this ‘old’ model open a new route to an effective vaccine.

## Methods

### Ethics statement

#### China

The housing conditions, experimental procedures and animal welfare of the monkeys used in the study were in strict accordance with the national guidelines for the Care and Use of Animals established by the Chinese National Animal Research Authority and applied by the Institutional Animal Care and Use Committee (IACUC) of the Kunming Institute of Zoology, Chinese Academy of Sciences (CAS). The experimental protocol was approved by the Ethics Committee of Kunming Institute of Zoology, CAS (ID SYDW-2011017). The study used six adult male rhesus macaques (*Macaca mulatta*) from the captive-breeding colony at the Kunming Primate Research Center, CAS. They were group-housed prior to the experiment but then singly after infection for faecal sampling purposes. The separate cages were arranged in one large room to allow the monkeys visual, olfactory and auditory interactions with each other. Food and water were available ad libitum and vitamins were provided. The animals were also provided with environmental enrichment, such as toys designed especially for monkeys, to promote psychological well-being. The design and execution of the study complied with the recommendations of the Weatherall report (2006) on “The use of non-human primates in research”, which specifically mentions the continuing requirement for their use in schistosome research.

#### UK

Procedures involving the vaccination of rats to raise antibodies against esophageal proteins were carried out in accordance with the UK Animals (Scientific Procedures) Act 1986, and authorised on personal (PIL 50/592) and project licences (PPL 60/4340) issued by the UK Home Office. The study protocol was approved by the Biology Department Ethical Review Committee at the University of York.

### Parasite exposure and sampling regime

The six adult male rhesus macaques used in the study had a mean age 9.67±0.82 years and a mean weight 7.98±0.85 kg at the outset. Cercariae of *S*. *japonicum* were shed from patent snails (*Oncomelania hupensis*) provided by the Jiangsu Institute of Parasitic Diseases (Wuxi, China), collected from the water surface using a bacteriological loop and placed on glass cover slips for infection. Rhesus macaques anaesthesized with ketamine hydrochloride (6 mg/kg body weight, Gutian Pharmaceutical Corporation, Fujian, China), were infected percutaneously with 600 cercariae, via the shaved abdominal skin for 30 minutes. Blood was obtained by intravenous sampling prior to infection and at 2-week intervals throughout the experiment up to perfusion (week 22), stood at room temperature for 1hour to clot, and kept overnight at 4°C to facilitate clot retraction before serum was recovered for storage at -20°C. The body weight of each monkey was also determined monthly. All animals were individually inspected daily. Those showing signs of diarrhoea were given oral dehydration therapy as required.

### Indirect estimates of infection intensity

Faecal samples were collected overnight at 2 week intervals from week 2. The number of eggs per gram of faeces (EPG) was determined from three individual samples/animal/time point, using both the Percoll technique [22] and the Kato-Katz method [23]. For Percoll, 250 mg fresh faeces were suspended in 3 ml phosphate buffer saline (PBS) and layered on top of 3 ml of 0.9% NaCl/ 60% Percoll (Sigma, Germany) solution. After centrifugation, the pellet was re-suspended in a small volume and suspension passed through a mesh sieve to exclude larger particles; the eggs in the flow-through were then counted under a microscope. For the Kato-Katz method, nine slides, each containing 41 mg of sieved stool, were prepared from three individual stool samples and examined by qualified technicians in a blinded manner. Soluble circulating anodic antigen (CAA), released into the bloodstream from the parasite’s gut, was measured using the up-converting phosphor lateral flow (UCP-LF) technology as previously described [24,25], with modifications. Briefly, serum was treated with 4% (wt/vol) trichloroacetic acid to remove proteins and antibody complexes. After centrifugation at 10,000xg for three minutes, the supernatant was mixed with an assay buffer containing an anti-CAA mouse monoclonal antibody conjugated to UCP reporter particles and incubated for 1 hour at 37°C. A lateral flow strip was placed into the mixture and chromatography was permitted to continue until strips were dry. Strips were read using a modified Packard Fluorocount meter, and the test line was normalized to the control line for each test strip; serum CAA levels were then expressed as the ratio of fluorescence counts of the test line/control line.

### Recovery of surviving worms

Perfusions to recover adult worms were performed 22 weeks after infection. In order to collect blood samples animals were first anaesthetized as described above. Heparin (Yi Cheng Bio technique Co. Ltd., Shanghai, China) (5000 units) was injected via the femoral vein afterwards and allowed to circulate for 5 minutes. The animals were then sacrificed by a further injection of sodium pentobarbitone. Portal perfusion was performed as described for the olive baboon with modifications [26]. Briefly, the thorax and abdominal cavity were opened and the aorta clamped above the bifurcation into the iliac arteries. The vena cava was clamped just before entry to the heart. A Foley balloon catheter was then introduced via an incision in the aorta and the hepatic portal vein was slit. RPMI-1640 medium (minus phenol red, Gibco, Life technologies) buffered with 10 mM HEPES (Invitrogen) was then infused into the aorta via a peristaltic pump, and the perfusate collected at the portal vein outlet. The worms were concentrated by gravity and washed in fresh RPMI-1640 medium plus 10 mM HEPES. After counting under a stereomicroscope, the worms from each macaque were divided into four groups and fixed for later processing. These four groups were used for morphological observations, immunocytochemistry, ultrastructure, and cryotomy, respectively. After fixation, worms were photographed in the different fixatives using a digital camera (Nikon D70 with manual macrolens and extension tubes to give x1.5 magnification) to record general morphological information and visual appearance. Body length as an index of well-being was measured from the resulting images of all worms using the ‘Analyzing digital images’ package (Lawrence Hall of Science). The amount of hemozoin pigment in the gut was also used as a second index but for females only. Based on their gut pigment, they were classified as: black, gut completely full from ovary to tail, plus traces in the anterior bifurcated section; intermediate, pigment incomplete in the posterior region; white, virtually no pigment anywhere.

Reference normal worms were recovered from C57BL/6 mice and rabbits (National Institute of Parasitic Diseases, Chinese Center for Disease Control and Prevention) six weeks after exposure to 40 and 1000 cercariae, respectively, fixed and photographed as above.

### Morphological analysis of survivors

Random samples of worms were fixed and stored in AFA (ethanol/40% formaldehyde/glacial acetic acid, in the ratio 85:10:5). Their morphology was visualised by staining for 30 minutes in Langeron’s Carmine [27], differentiation in 70% acid ethanol until background stain had disappeared, clearing in Histoclear (National Diagnostics UK, Hessle, UK), and mounting in DPX (VWR International Ltd, Lutterworth, UK).

High magnification pictures of the ovaries of carmine-stained worms were obtained using a MZ16F stereo microscope (Leica Microsystems, Milton Keynes, UK) fitted with a Spot RT3cooled CCD camera (Diagnostic Instruments Inc, Sterling Heights, MI). The surface area in cross section of ovaries was calculated using length and width measurements from digital images, and the formula π a b, where a and b are the minor and major axes of an ellipse.

Total eggs in the uterus of 34 females from rhesus macaques were independently counted directly under a compound microscope by two people. The uterine egg complement of females from rabbits and mice was determined after length measurement, by digesting singly in 10% KOH at 37°C for 1 hour to dissolve the body tissue before counting.

### Immunocytochemistry

For immunocytochemistry, intact adult worms were fixed and permeabilized using the protocol of Mair et al. [28]. The scope of this investigation was limited by the number of worms recovered from each monkey. Briefly, they were fixed for four hours in 4% formaldehyde and then incubated in permeablising fluid (PBS containing 0.1% Triton X-100, 0.1% BSA and 0.02% sodium azide; antibody diluent solution, AbD) overnight at 4°C. Subsequent steps were carried out, with shaking, at 4°C in AbD.

To detect the presence of host antibodies, permeabilized worms recovered from macaques were incubated with FITC-labelled rabbit anti-monkey IgG (H+L;Sigma-Aldrich, Poole, UK for two days at 4°C and given extensive washes in AbD before viewing. To explore details of intrinsic bound antibody in the esophageal lumen, worm head cryosections were prepared as described by Li et al [18] and reacted with 1:100 dilution of FITC- labelled rabbit-anti monkey IgG. The musculature and nuclei were visualized by staining of f-actin with a 1:100 dilution of AF555-conjugated phalloidin (Invitrogen, Molecular Probes) plus 4',6-diamidino-2-phenylindole (DAPI; diluted in 1:600 in 10% Normal Goat serum in PBS; Sigma-Aldrich, Poole, Dorset, UK), respectively, for 30 minutes.

Although only one MEG has so far been described in *S*. *japonicum*, an analysis of genes highly up-regulated in the Day 3 skin schistosomulum of *S*. *mansoni* has provided pointers to other genes potentially expressed in the esophageal region [29]. We were thus able to find predicted sequences for SjMEGs 4.2, 8.2, 9, 11 and14, and SjVAL-7, by searching the NCBInr database using the homologues of known or suspected esophageal gland proteins from *S*. *mansoni* [17,29]. To investigate the localization of these six proteins, antibodies were raised by vaccinating rats either with the purified recombinant expressed in a modified pET28a system (SjVAL-7 and SjMEG-8.2) or a synthetic peptide (S2 Table) derived from the parent protein sequence and coupled via a C-terminal cysteine to carrier ovalbumin. The two recombinant proteins were purified from *E*. *coli* lysates after induced expression, using nickel-column affinity chromatography. The homogeneity of the preparations was confirmed by 1D SDS-PAGE electrophoresis and the amino acid composition by tandem mass spectrometry. A 100 μg sample in 0.1 ml phosphate buffered saline (PBS), emulsified in 0.1ml complete Freund’s adjuvant (Sigma-Aldrich), was administered subcutaneously to rats on the back of the neck, with two subsequent boosts at 3-week interval with conjugates emulsified in incomplete Freund’s adjuvant, before a terminal bleed at week 8. The antibody titres of individual rats were determined by ELISA using plates coated with bovine serum albumin-conjugated synthetic peptides or purified recombinant proteins. Permeabilized *S*. *japonicum* worms were reacted with the six rat antibodies at 1:1000 dilution in AbD containing 10% normal goat serum and washed extensively in AbD before localization using Alexa Fluor (AF) 488-labeled goat anti-rat antibody at 1:100 dilution (Invitrogen). To explore the correlation between intrinsic IgG distribution and localisation of known secreted proteins from esophageal glands, worms previously probed with anti-monkey IgG were reacted with seven rat primary antibodies (the above six plus anti-MEG-4.1 Ab) before localization using AF647-labeled goat anti-rat antibody (Invitrogen) at 1:100 dilution. The musculature and nuclei were visualised as described above for cryosections, except that permeabilised worms were incubated overnight with a 1:100 dilution of phalloidin.

Optical slices were obtained using a LSM-710 confocal microscope (Zeiss, Cambridge, UK). Imaging conditions were as follows: DAPI: 405 nm diode laser with 405 main beam splitter (MBS); FITC/AF488: 3 mW argon laser with 488/561/633 MBS; Phalloidin AF555: 561 nm diode laser with 488/561 MBS; AF647: Helium/Neon laser with 488/561/633 MBS; Langeron’s carmine: 561 nm diode laser with 488/561 MBS.

### Transmission electron microscopy and scanning electron microscopy

The structure of the esophageal region in representative worms from self-cured rhesus macaques, showing little or no gut pigment, was examined by TEM and SEM exactly as described by Li et al [18].

### Antibody responses

Levels of IgG antibodies against soluble adult worm proteins (SWAP) and soluble egg antigens (SEA) were determined by ELISA, as described previously [14]. ELISA plates were coated at an Ag concentration of 1μg/well and all serum samples diluted 1:1600 to allow between-time and-sample comparisons. Wells were probed with peroxidase-labelled rabbit anti-monkey IgG (Sigma-Aldrich), diluted 1:2000, colour developed using TMB substrate (Sigma-Aldrich) and absorbance read at 450 nm.

To detect the presence of antibodies against SjMEGs 4.1, 4.2, 8.2, 9, 11and 14, and SjVAL-7 antigen, we coated ELISA plates with each synthetic peptide conjugated to a carrier protein BSA or with recombinant proteins (for SjMEG-8.2 and VAL-7) at 1μg/well and performed the ELISAs as above.

### Statistics

Differences between normally distributed variables were tested for significance using Student’s t test. The significance of trends in antibody titre against SEA and SWAP antigen preparations over selected sections of the time-course was assessed by linear regression using the r^2^ value as the indicator. Fluctuations in monkey body weight after infection were assessed using both Student’s t test and the Wilcoxon Signed-Rank Test on paired data sets.

## Results

### Rhesus macaques eliminate the adult *S*. *japonicum* worm population by a self-cure process

**Fig 1:**
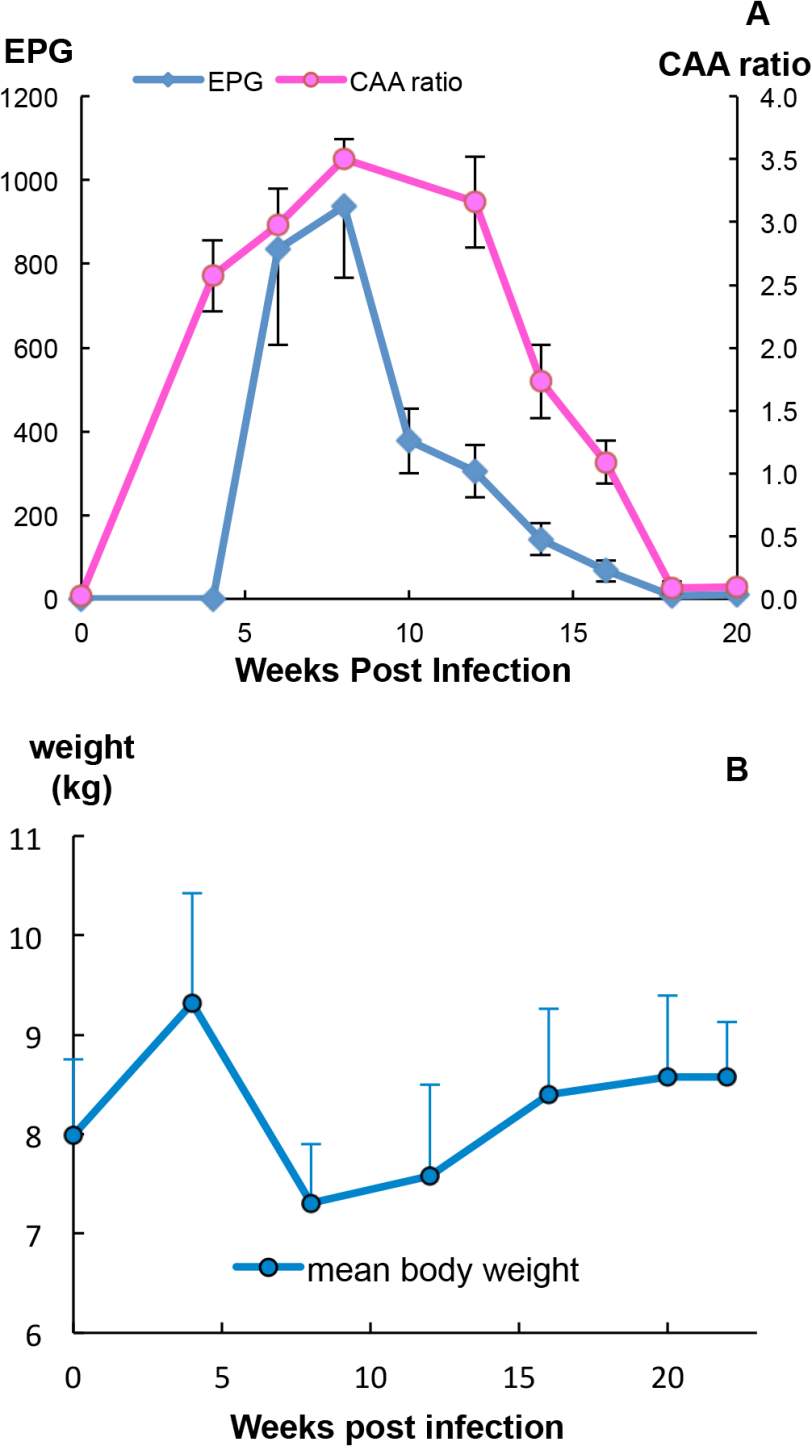
The profile of the self-cure process. (A) Fecundity of the worm population, revealed by fecal egg output reached its peak at week 8, dropped sharply thereafter and then more slowly reached zero, at the end of the study. The level of soluble circulating anodic antigen (CAA, expressed as the ratio of test/control) released in the bloodstream of rhesus macaques, an indicator of worm blood feeding and gut function, was already high at week 4 but started declining after week 12. Values are mean + or—SE, n = 6 animals. (B) The body weight of monkeys increased during the first 4 weeks after infection, declined thereafter reaching the minimum value at week 8 but then gradually recovered towards the weak 4 peak weight by the end of the study. Values are mean + or–SD, n = 6 animals.

The status of the worm population in the rhesus macaques over the 22 week time course was evaluated by two surrogate measures of worm burden: egg number in the faeces, and the level of soluble circulating anodic antigen (CAA) derived from the worm gut in the bloodstream. Faecal eggs were detected as early as 5 weeks after infection with cercariae. The mean value for egg output peaked at week 8 at 900 eggs per gram of faeces (EPG, Fig 1A; range from 480–2000). However, it then dropped sharply to approximately 300 at week 10 and continued to decline thereafter but at a much slower rate. In two animals, egg excretion reached zero by the end of the study. The profile of CAA was similar to that of egg output except that the levels were already high by four weeks, indicating that blood feeding was well underway (Fig 1A). The CAA level rose steadily as worms reached maturity, with mean values plateauing at week 8 before declining linearly from week 12 onwards. There was very little CAA detected at the last two sampling times. We estimated the worm burden in each monkey at peak by combining the EPG values with published data on egg output per female, the fraction of eggs excreted into the faeces [30] and daily faecal production by macaques [31] (S1 Table). The estimated burden ranged from 136 to 560 worms, equating to between 23% and 93% of applied cercariae. In contrast, at perfusion there was a wide discrepancy in the number of worms recovered (S1A Fig) with burdens ranging from 15 to 112, equivalent to between 2.5% and 18.7% of applied cercariae.

Unexpectedly the monkeys increased their body weight by a mean of 17% over the first 4 weeks (t-test, P<0.05) in spite of being exposed to 600 schistosome cercariae (Fig 1B), presumably due to absence of food competition since they were now singly housed. However, a marked decline in body weight, coincident with the onset of egg deposition, was observed between 4 and 8 weeks to a mean of 79% of the week 4 weight (t-test, P<0.01). All monkeys suffered from severe diarrhea and inappetence during this period. A gradual recovery occurred thereafter so that by week 16 the group mean was not significantly different from the week 4 peak weight. The non-parametric Wilcoxon Signed Rank Test for the significance of percentage changes in weight over the time course gave the same results as the t-test (weeks 0–4, 4–8 and 4–12, P<0.05; weeks 4–16 and 4–20, not significant).

### Surviving worms recovered at 22 week post-infection show evidence of physiological malfunction

**Fig 2:**
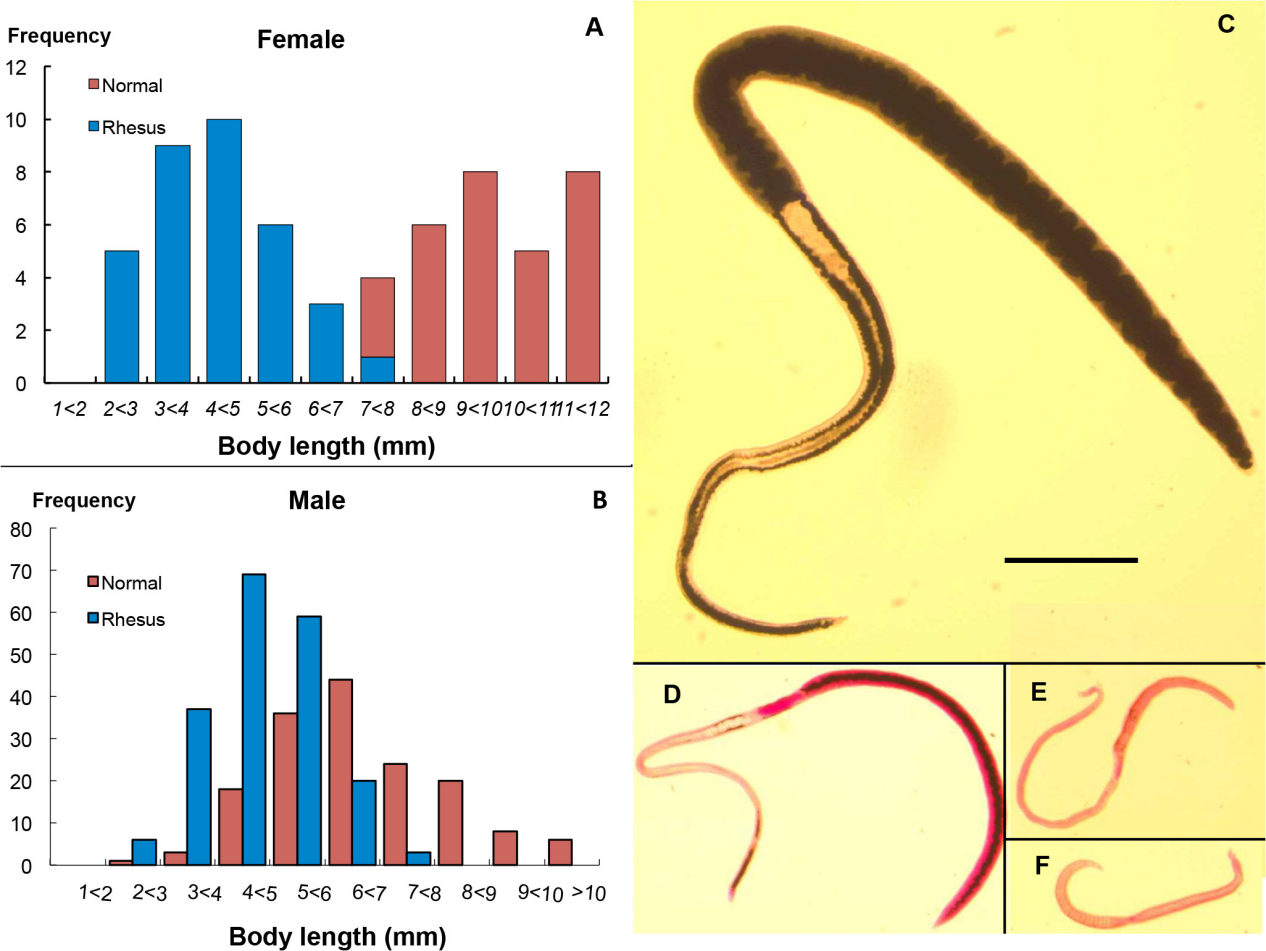
Females are more susceptible to immunological pressure than males in rhesus macaques. The body lengths of females (A) and males (B) from rhesus macaques, were shorter than their equivalents from rabbits, but the two male populations overlapped considerably (B) whilst the females did not (A). The detailed difference in females from the two hosts, a mature female from a rabbit (C) and three from rhesus macaques (D to F) all to scale (bar = 1mm), are compared. (C) The rabbit worm has black hemozoin pigment present along the whole length of the body. Females from monkeys were smaller and showed different levels of pigment in the gut: (D), Black group were full of pigment from ovary to tail, plus traces in the anterior; (E), Intermediate group had incomplete pigment only in the posterior region; (F), White group had virtually no pigment anywhere.

In appearance, the worms recovered from rhesus macaques 22 weeks post-infection differed distinctly from those that developed in the rabbit permissive host (S1B and S1C Fig). Both males and females were smaller in size compared with their normal equivalent from permissive hosts. Female body length ranged from 2.3 to 7.48 mm, the majority of them being shorter or much shorter than controls; indeed there was minimal overlap in the two populations (t-test, P<0.01) (Fig 2A). The size of males from rhesus macaques was also smaller than those from permissive hosts (t-test, P<0.05) (Fig 2B), although there was considerable overlap in the two frequency distributions. Females were generally paler (S1B Fig) than those from rabbits, which are always black along the length of their bodies (S1C Fig and Fig 2C). When females were classified by the amount of hemozoin in their guts, approximately 37% came within the black category (Fig 2D and S1A Fig) although not strictly comparable to normal worms (Fig 2C), both in respect of body size and pigment intensity. The remaining two thirds of females had intermediate amounts (Fig 2E and S1A Fig) or no pigment (Fig 2F and S1A Fig) in their guts, suggesting they either had difficulty ingesting blood for some time or had stopped feeding altogether and were starving to death. The average body length for black, intermediate and white groups was 5.29 mm, 4.75 mm and 4.15 mm, respectively (all significantly different; t-test, P<0.05), indicating that the parameter correlated with the amount of blood consumption. Indeed, the most pallid females at perfusion looked ‘sick’ or moribund. The mean female:male ratio of surviving worms in rhesus macaques was 0.751:1, less than that in rabbits (0.888:1) (t-test, P<0.05), suggesting females are more susceptible than males to elimination in rhesus macaques.

### The reproductive capacity of surviving females was diminished

**Fig 3:**
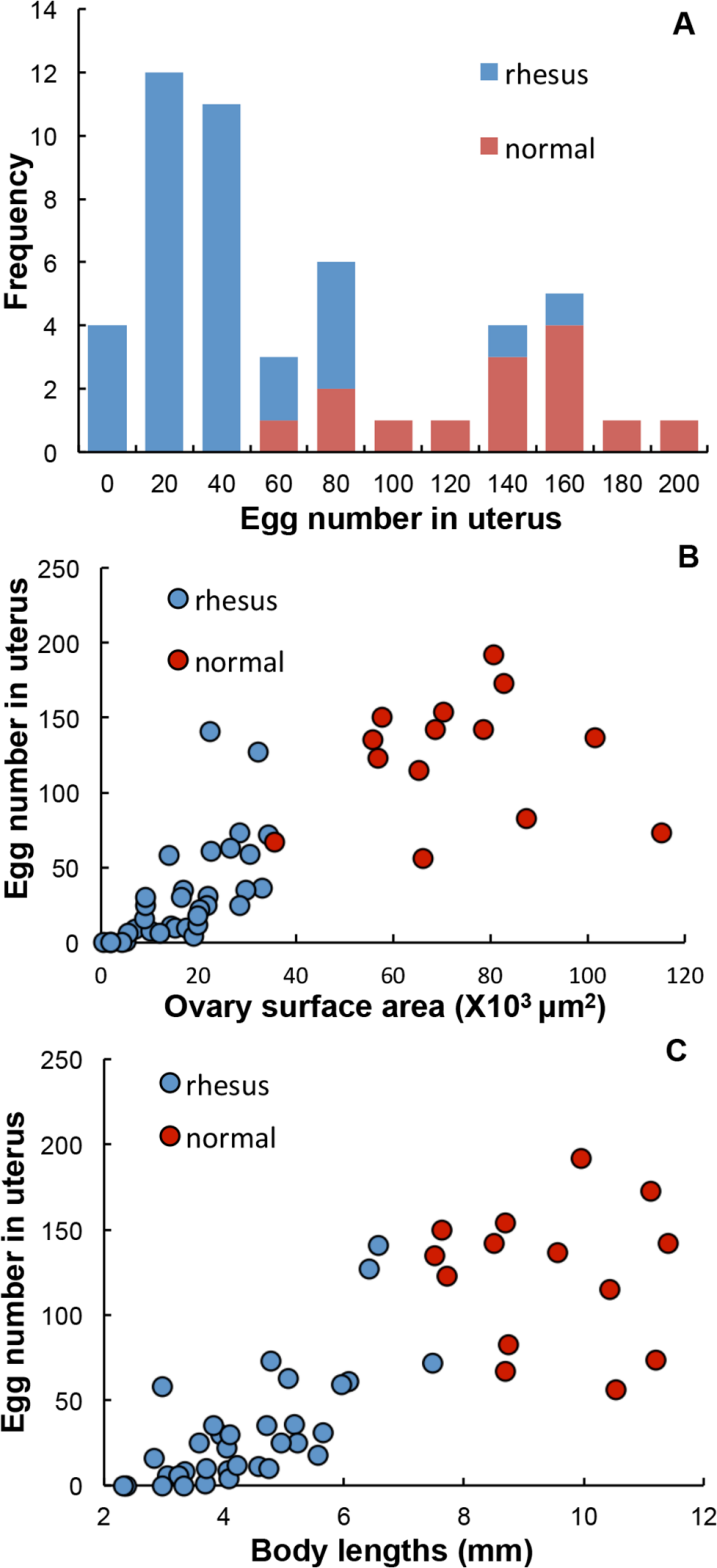
Reproductive capacities of female worms from rhesus macaques were diminished. (A) Egg number in the uterus of females from two populations. Those from rhesus macaques (blue) had a wide range from zero to 141, the majority having from 20 to 40 eggs. Those from permissive rabbit and mouse hosts also had a wide range of egg numbers, but with the majority between 140 and 160. (B) and (C) illustrate that ovary size and body length correlate positively with egg number in the uterus, with almost no overlap. Females from rhesus macaques (blue), had smaller ovaries (B), shorter bodies (C) and fewer eggs, compared to control females (red).

Unlike *S*. *mansoni* and *S*. *haematobium*, mature *S*. *japonicum* females that develop in permissive hosts have a robust reproductive capacity, with large numbers of eggs in the uterus and a total egg output up to 2000 eggs/female/day [30]. The egg number in the uterus of the normal mouse and rabbit worms examined was not significantly different (t-test, P> 0.05) so the data were pooled; it ranged from 56 to 192 (Fig 3A), (median = 135; mean = 124 ± SE 37). By contrast, surviving females from rhesus macaques generally had many fewer eggs in their uterus, 77% of them with <40 (Fig 3A), (median 23.5; mean = 31.4 ± SE 5.8; t-test, P<0.0001). Nevertheless, there was a wide range between individuals: four females had no eggs while two had more than 100, signifying that they retained their reproductive capacity. Ovary surface area was linearly related to number of eggs in the uterus (Fig 3B), with the survivors from rhesus macaques all having smaller ovaries and fewer eggs in their uterus, than those from the permissive hosts with large ovaries and many eggs (areas significantly different, t-test, P<0.0001). The linear relationship was even more prominent between egg number and body length (Fig 3C). Indeed, the three categories of females from rhesus macaques, although varying in their level of gut pigment, all showed other marked changes in their reproductive system (S2 Fig). The vitelline cells in vitelline ducts were smaller and more widely spaced (S2A Fig) whilst the oviduct was inflated (S2C Fig), compared with controls (S2D Fig). Massive numbers of spermatozoa were adherent to regions of oviduct in both ‘white’ and ‘black’ females (S2A and S2B Fig), waiting to fertilise an egg. Apparently insemination continued but egg production was reduced or terminated.

### The mechanism of vesicle release in the anterior esophagus appears disabled

**Fig 4:**
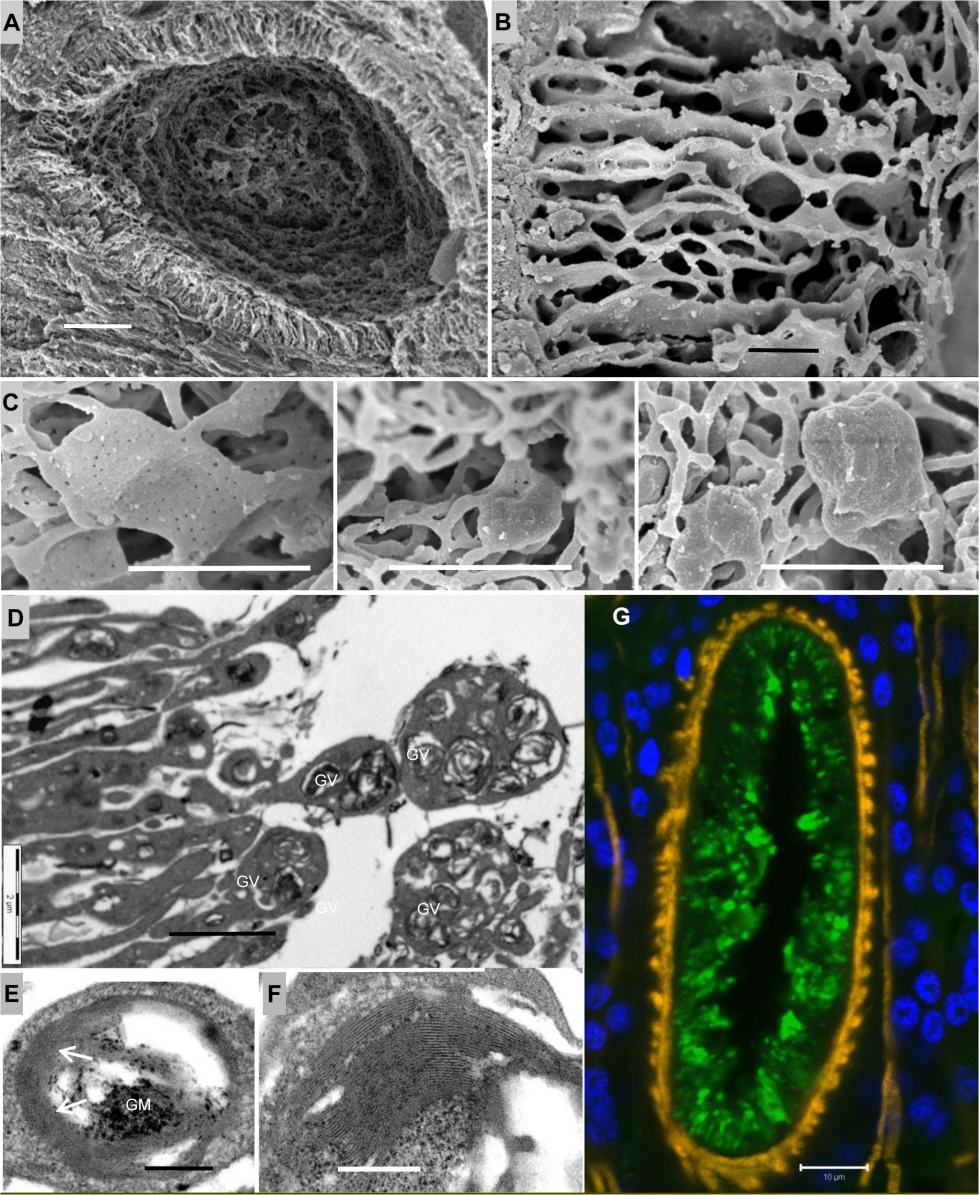
Vesicle release in the anterior esophagus of worms from rhesus macaques is disrupted. (A) SEM of a transverse thick section of a worm head, displaying an internal view of the anterior esophagus. The lining corrugations appear short and shrunken; (B) High magnification SEM showing the honeycomb appearance of anterior corrugations, which look more fragile than in normal worms; (C) Three to-scale SEM images of flat plates of cytoplasm in the fringe of the anterior lining, obscured by a deposit of material. Their surface pits varied from ‘largely visible’ (left), through ‘barely seen’ (middle), to ‘completely obscured’ (right), giving the whole flat plate a lumpy spheroidal appearance. (D) TEM of the luminal edge of the anterior lining, showing its tips greatly expanded beyond the normal situation, and containing giant vesicles (GV). (E) TEM of a giant vesicle showing its contents include both granular material (GM) and membrane stacks (arrowed). (F) High magnification TEM reveals that the membrane stacks comprise alternating light and dark bands of material, resembling the membranocalyx-coating of the esophageal surface lining. (G) Confocal image of a transverse section of a worm head stained with AF488 labeled anti-monkey IgG (green), showing the focal and punctuate staining in the anterior lining; both intense spheroids of IgG staining and smaller, fainter spots are evident. DAPI-stained nuclei are blue and phalloidin-stained muscle is orange. Scale bars: 10 μm (A, G), 2 μm (B, C, D), 200 nm (E, F).

Having established that surviving worms had mostly ceased blood feeding, we wished to discover if there were changes in the cellular structure of feeding-related organs. The anterior esophagus in *S*. *japonicum* functions as a secretory organ, delivering the contents of light vesicles to the lumen where they can interact with incoming blood [18]. In worms from permissive mouse hosts the narrow cytoplasmic corrugations that line the compartment terminate in threads of cytoplasm that create a mesh-like structure in the center (S3 Fig, Part 1C). However, in rhesus worms SEM revealed that these corrugations were shorter and the fringe of cytoplasmic threads was much reduced, losing its mesh-like qualities (Fig 4A). The individual corrugations appeared more fragile, with fenestrations giving a honeycomb appearance (Fig 4B versus S3 Fig, Part 1D). In addition, the minute pits on the flat plates of cytoplasm located within the fringe were obscured to varying degrees by a deposit of material, to become rounded, lumpy spheres (Fig 4C versus S3 Fig, Part 1F), with an average size of 1.54 x1.20 μm^2^.

The tips of corrugations in mouse worms contain clusters of light vesicles in males and a single vesicle in females (dimensions: surface area of a cluster, ~1 μm^2^; single vesicle, 0.43x 0.31 μm^2^) (S3 Fig, Part 2A-2D). In contrast, TEM revealed that the corrugation tips in rhesus worms had a greatly enlarged surface area, up to 5 μm^2^, mean size being 3.5 μm^2^ (Fig 4D). They contained more vesicles and the individual vesicles were larger (0.78 x 0.63 μm^2^). Indeed the largest rhesus worm vesicle was 1.13 x 1.02 μm^2^, which translated into volume made it 28 times larger than the average mouse worm vesicle (Fig 4D versus S3 Fig Part 2A). The contents of the enlarged rhesus worm vesicles were proportionately similar to those from mouse worms but the quantities of both granular material and membrane stacks were amplified (Fig 4E versus S3 Fig Part 2F-2G). At high magnification the membrane stacks comprised alternating light and dark bands of material that would normally be destined to refresh the surface membranocalyx of the anterior esophagus (Fig 4F). The central core of granular material comprised dark specks scattered over a lighter ground substance, indicating two classes of constituent. Finally, confocal microscopy on the cryosections of the anterior esophagus revealed intense oval deposits of IgG on the lining (average size 2.04 x 1.14 μm^2^), plus a background of smaller, fainter spots (Fig 4G).We also detected fibrin in the oval deposits (S4 Fig), although blood clot formation does not normally occur in the esophagus and staining was incompletely superimposed on the pattern of rhesus IgG.

### The posterior esophagus in starving worms has an altered morphology that may affect blood processing

**Fig 5:**
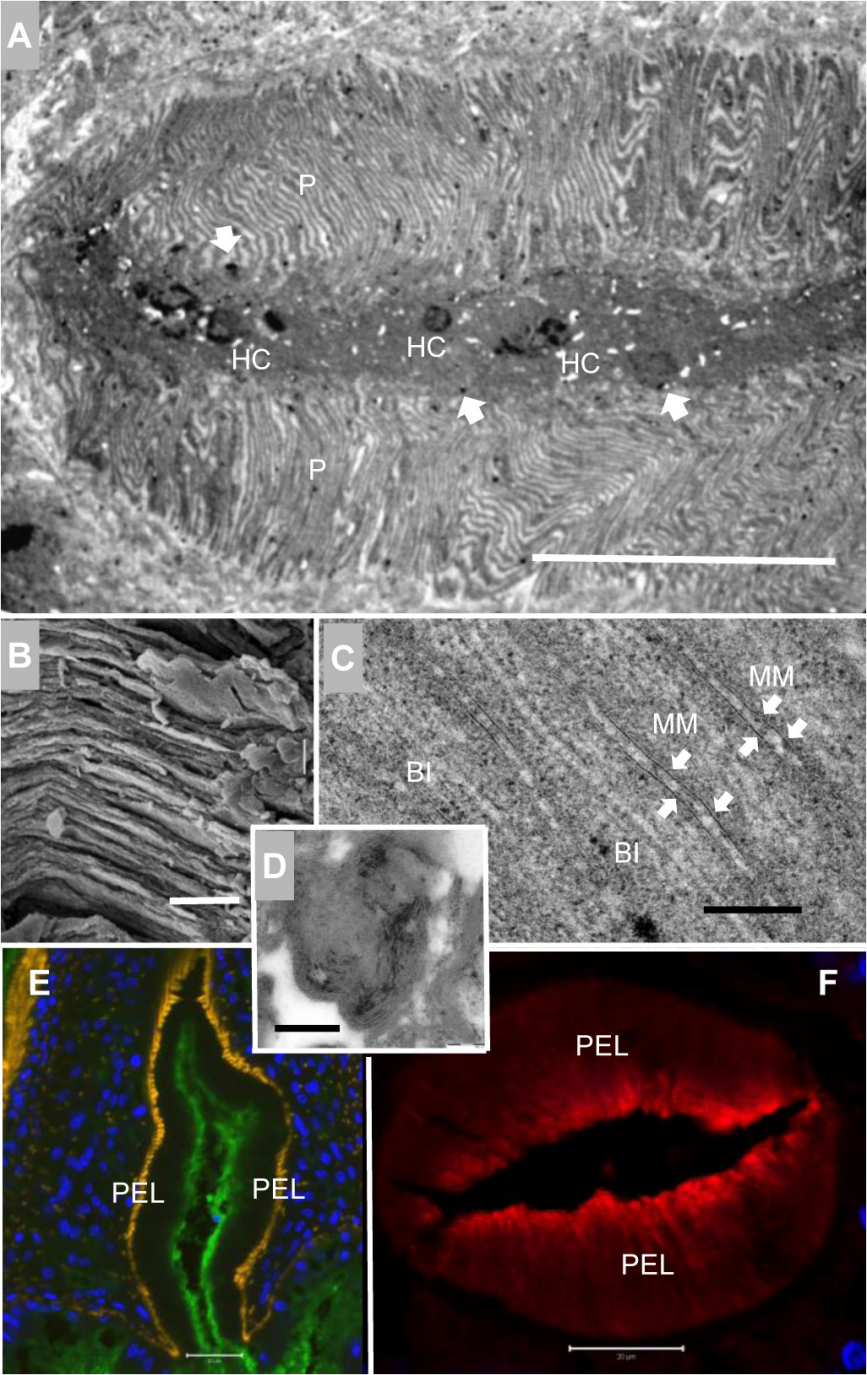
Host antibody binds to the posterior esophagus which shows an altered cellular morphology. (A) TEM image of the posterior esophagus, showing the luminal plates (P) tightly packed and closely adherent. The lumen appears congested, occluded by debris and host cells (HC) in the center; electron dense structures (arrowed) are also visible around the edges of the debris. (B) SEM image of the plates in the posterior esophagus, showing that intervening spaces are largely absent. (C) TEM image at high magnification reveals the parallel tramline appearance of posterior esophageal plates. Where two adjacent plates are particularly closely adherent, membranous material is visible (MM, arrowed) bound to the luminal surface. The two lighter lines in the center of each plate denote the basal invagination (BI). (D) High magnification image of electron dense structures revealing they are aggregates of membranous material. Confocal images of male worm heads: (E) a longitudinal section, stained with AF488 labeled anti-monkey IgG, and (F) a transverse section, stained with Cy3 labelled anti-monkey IgG. (E) is counterstained with DAPI (blue) and phalloidin (orange) to highlight the nuclei and musculature and (F) with DAPI only. Both images reveal that intrinsic antibody (green in E, red in F) has bound to the post esophageal lining (PEL). The luminal edges of lining plates are strongly positive whilst the surface of the plates is more weakly stained. Scale bars: 20 μm (A, E, F), 2 μm (B), 200 nm (C, D).

*S*. *japonicum* worms recovered from permissive hosts almost always have a plug of tethered leucocytes in the lumen of the posterior esophagus around which incoming blood flows (S5A Fig). In contrast, the posterior esophageal lumen of starving rhesus worms was always completely occluded by a mass of tissue debris in which the remnants of ingested host leucocytes (nuclei) were visible (Fig 5A). The condition of the cytoplasmic plates that projected into the lumen was also altered. Normally these are separated by spaces in which the aggregated secretions are visible (S5B Fig). In rhesus worms the plates appeared closely adherent with spaces largely absent (Fig 5A and 5B). Indeed, at higher magnification the spacing between the outer surfaces of adjacent plates averaged 17 nm over long stretches, giving the appearance of tramlines running in parallel (Fig 5C). Electron-dense structures (Fig 5A) were also visible around the edges of the debris (arrowed). Close inspection revealed these to be aggregates of membranous material or even intact macro vesicles, presumably derived from the secretions of the anterior esophagus (Fig 5D). Immunocytochemistry showed intrinsic host antibody bound to the lining of the posterior esophagus, with the luminal edges being strongly positive, whilst the plate sides stained more weakly (Fig 5E and 5F). All these changes are evidence that worm starvation is the result of an occluded esophagus lumen with host antibody the causative agent.

### Antibodies target the adult worm esophagus

**Fig 6:**
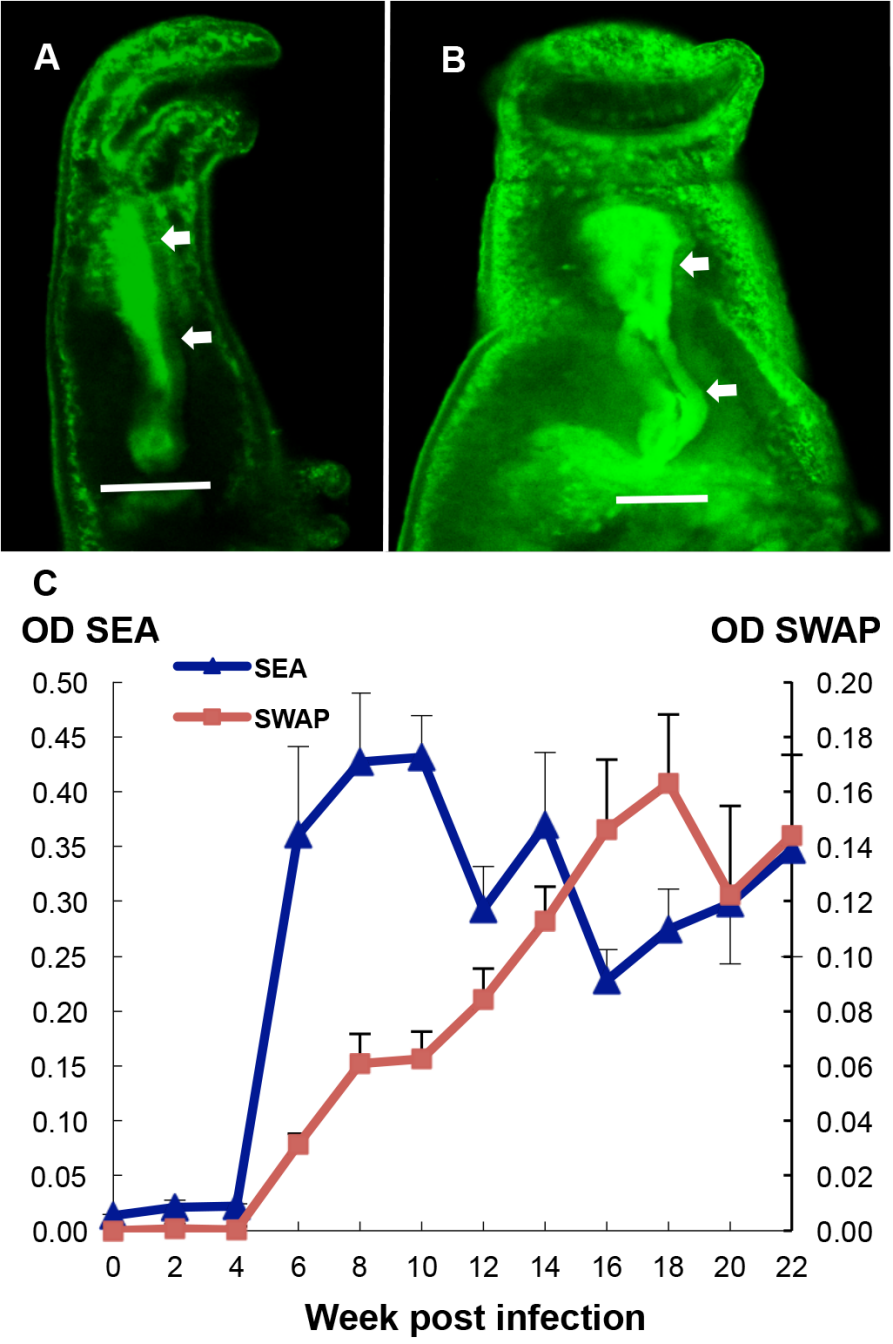
Antibody targets the worm esophagus and shows distinct response patterns to worm and egg antigens. Permeabilized whole worms recovered from rhesus macaques, (A) female and (B) male, reacted with FITC-labeled anti-rhesus IgG, showed intrinsic antibodies (green) bound to the tegument, particularly in the head region, but more strikingly along the whole length of the esophageal lumen (arrowed). (C) Time course of antibody response against soluble worm antigen (SWAP), and egg antigen (SEA) preparations. Binding of antibody to both preparations was barely detected during the first 4 weeks. Thereafter the anti-SEA response rose dramatically to reach a high plateau at week 8, but the decline from week 10 onwards was not significant. In contrast, anti-SWAP reactivity rose only slowly from week 4, with its peak appearing as late as week 18. Scale bars: 50 μm (A), 20 μm (B).

In order to discover which worm structures were targeted by rhesus IgG, we reacted permeabilized intact adult males and females with FITC-labeled second antibody. The tegument of both sexes was strongly positive, particularly around the anterior (Fig 6A and 6B). However, sections of the alimentary tract were also positive, the anterior esophageal lumen and lining being especially intense, and to a lesser extent the posterior esophagus and the transverse gut (Fig 6B). These observations strongly suggest that the esophagus is a major ‘battlefield’ between the worms and the host, whilst not ruling out the other targets.

We attempted to develop an esophageal antigen preparation by physical enrichment, cutting off male worm heads. However, proteomic analysis revealed that the dominant constituents were still the abundant cytosolic and cytoskeletal proteins present in SWAP (S3 Table). We therefore monitored the longitudinal antibody profile to worm and eggs using crude soluble antigenic extracts from the two life-cycle stages. No response against SEA was observed over the first four weeks but by week 6 the antibody titer had risen dramatically (Fig 6C) as a consequence of egg deposition in tissues. The level continued to rise, reaching the peak value at week 10 but the apparent erratic decline thereafter was not statistically significant (regression r^2^ = 0.048, P>0.05). As with the response to eggs, there was no detectable antibody to SWAP over the first four weeks. However, unlike the response to eggs, the IgG against worm antigens increased steadily from week 4 onwards, before reaching a plateau as late as week 18 (Fig 6C) (regression r^2^, weeks 4 to 18 = 0.5, P<0.0001; weeks 18 to 22, r^2^ = 0.01, P>0.05). The higher OD values for the anti-egg compared with the anti-worm reactivity emphasized the much stronger response that the host made to eggs and their products than to worm constituents.

### MEG and VAL proteins are exclusively expressed in the posterior esophageal gland

**Fig 7:**
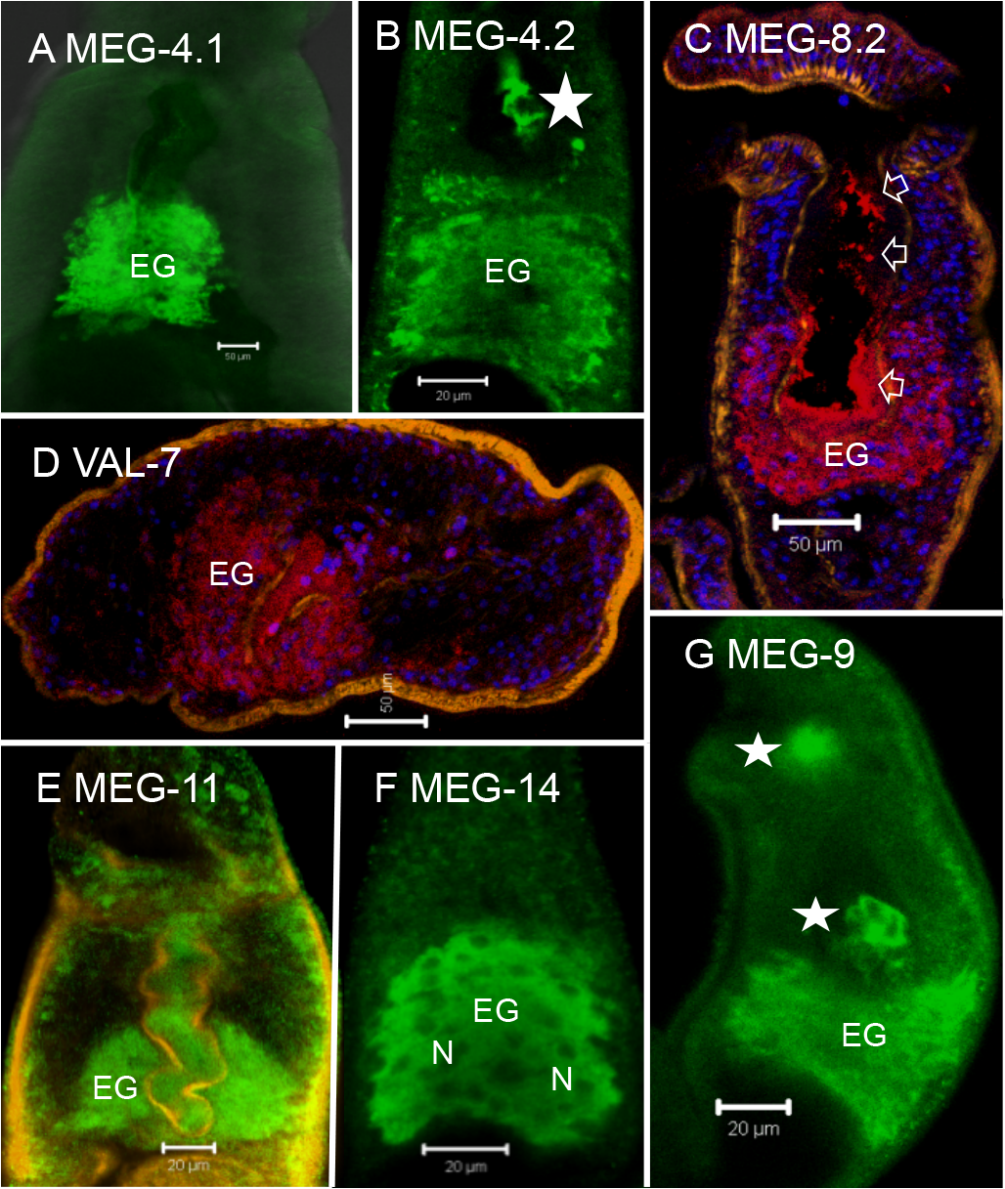
*S*. *japonicum* MEG and VAL proteins are localized to the posterior esophageal gland. Localization of target proteins on permeabilized whole worms (green, A, B and E to F), and cryosections (red, C and D) by immunocytochemistry, showing that all seven proteins, (A) MEG-4.1, (B) MEG-4.2, (C) MEG-8.2, (D) VAL-7, (E) MEG-11, (F) MEG-14 and (G) MEG-9 were solely expressed in the esophageal gland (on C & D, nuclei are blue and muscle is orange). Only the cytoplasm of the gland cell bodies (EG) was strongly positive, whilst the individual nuclei (N in Fig 7F) appeared as dark unstained spheres. The association of at least three proteins (MEG-4.2, MEG-8.2 and MEG-9) with host leucocytes in the lumen (starred, B and G) and the oral cavity (starred, G), or by a coating on both anterior and posterior esophageal linings (arrowed, C), confirmed that they were secreted. Scale bars: 50 μm (A, C, D), 20 μm (B, E, F, G).

Although no MEGs are annotated in the *S*. *japonicum* genome database we were able to identify cDNA sequences for seven of these genes by BLAST searching of the NCBInr database. Antibodies were successfully raised against synthetic peptides from SjMEGs 4.1, 4.2, 9, 11 and 14 and affinity-purified recombinant proteins for SjMEG-8.2 and SjVAL-7 (S6 Fig). Immunocytochemistry on either permeabilized whole worms or cryosections revealed that all seven proteins were expressed in the posterior esophageal gland of the adult *S*. *japonicum* worms (Fig 7A–7G). The pattern of staining was identical in each case, with the cytoplasm of the cell bodies being strongly positive, whilst the individual nuclei appeared as dark holes most clearly visible in Fig 7F. The secretion of the SjMEGs 4.2 and 9 was evident from their detection on host cells in the esophageal lumen (Fig 7B and 7G). The staining pattern for SjMEG-8.2 on cryosections of the esophagus revealed its presence both on the posterior and the anterior lining (Fig 7C).

### Esophageal gland secreted proteins are targets of serum from self-cured animals

**Fig 8:**
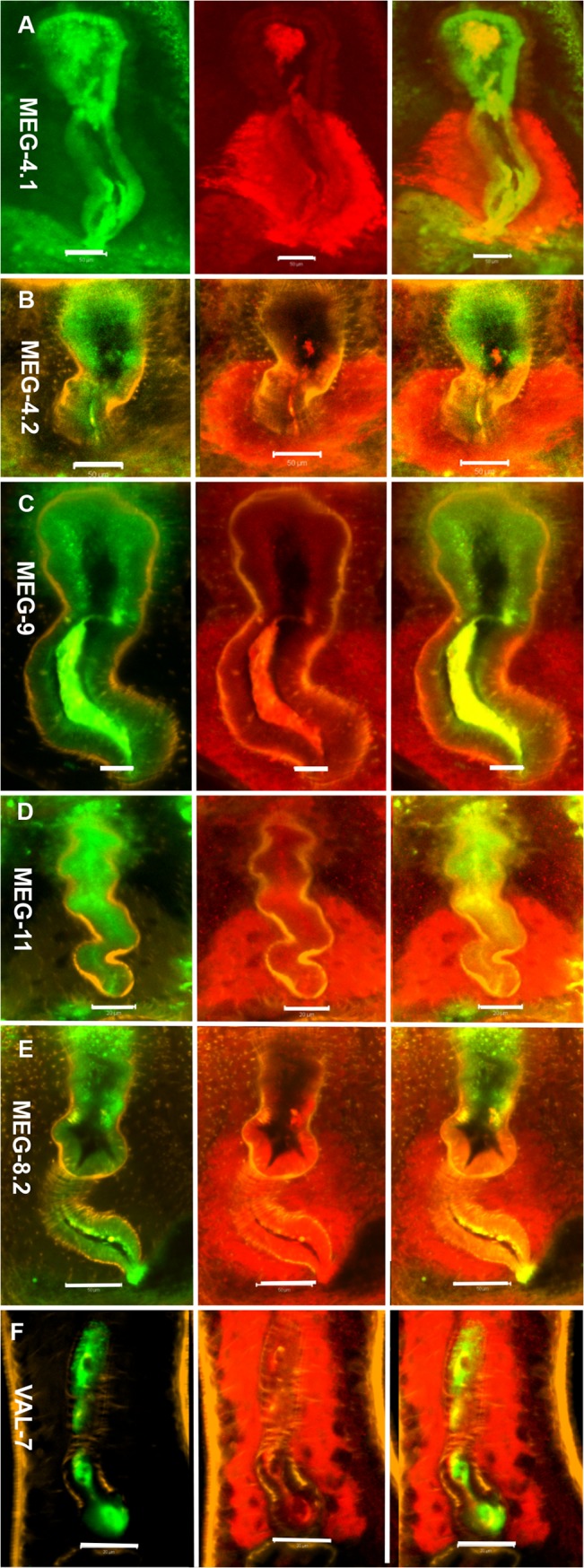
Host intrinsic antibody and esophageal gland proteins are co-localised. Immunocytochemistry on permeabilized whole worms from rhesus macaques revealed the localization of host intrinsic antibody (green), six esophageal proteins of *S*. *japonicum* (red) and worm muscle (orange). (A) MEG-4.1, (B) MEG-4.2, (C) MEG-9, (D) MEG-11, (E) MEG8.2 and (F) VAL-7. The triplet images display the distribution of host IgG in the first column, the esophageal proteins in the second, and the two overlaid in the third where the lemon yellow color indicates areas of co-localization. The pattern differed for individual esophageal proteins. Colocalization was only partial in MEG-4.2 (B), as free proteins (red) were visible in the anterior esophageal lumen. MEG-4.1 (A), MEG-9 (C) and MEG-11 (D) showed almost complete colocalization with the IgG and protein superimposed, and no free protein visible in the lumen. MEG-8.2 (E) colocalized with IgG in the posterior lining whilst VAL-7 (F) was present only in the posterior esophageal lumen. Scale bars: 50 μm (A, B, E), 20 μm (C, D, F).

As a pointer to immune reactivity we determined the extent of co-localization between host IgG in the esophagus and the presence of potential target proteins. A caveat was that such observations were only possible on worms recovered from high burden monkeys, which were likely to be the least reactive to worm secretions. In the third image of each triplet, a lemon yellow color indicated co-localization whereas regions of the lumen that remained red indicated that a given protein was not likely to be a target of IgG. In the case of MEGs 4.1, 9 and 11 proteins, IgG appeared to be almost completely superimposed with no free protein visible, suggesting they were likely targets (Fig 8A, 8C and 8D). Similarly, MEG-8.2 protein in both posterior and anterior esophageal lumen, and on the lining, was completely superimposed by IgG (Fig 8E). For VAL-7, the co-localization between IgG and protein appeared in the posterior esophagus (Fig 8F). However, whilst MEG-4.2 and IgG staining was superimposed in the posterior esophageal lumen (Fig 8B), free MEG-4.2 was visible in the anterior esophagus indicating it was not associated with IgG. No colocalization of IgG and MEG-14 was observed.

**Fig 9:**
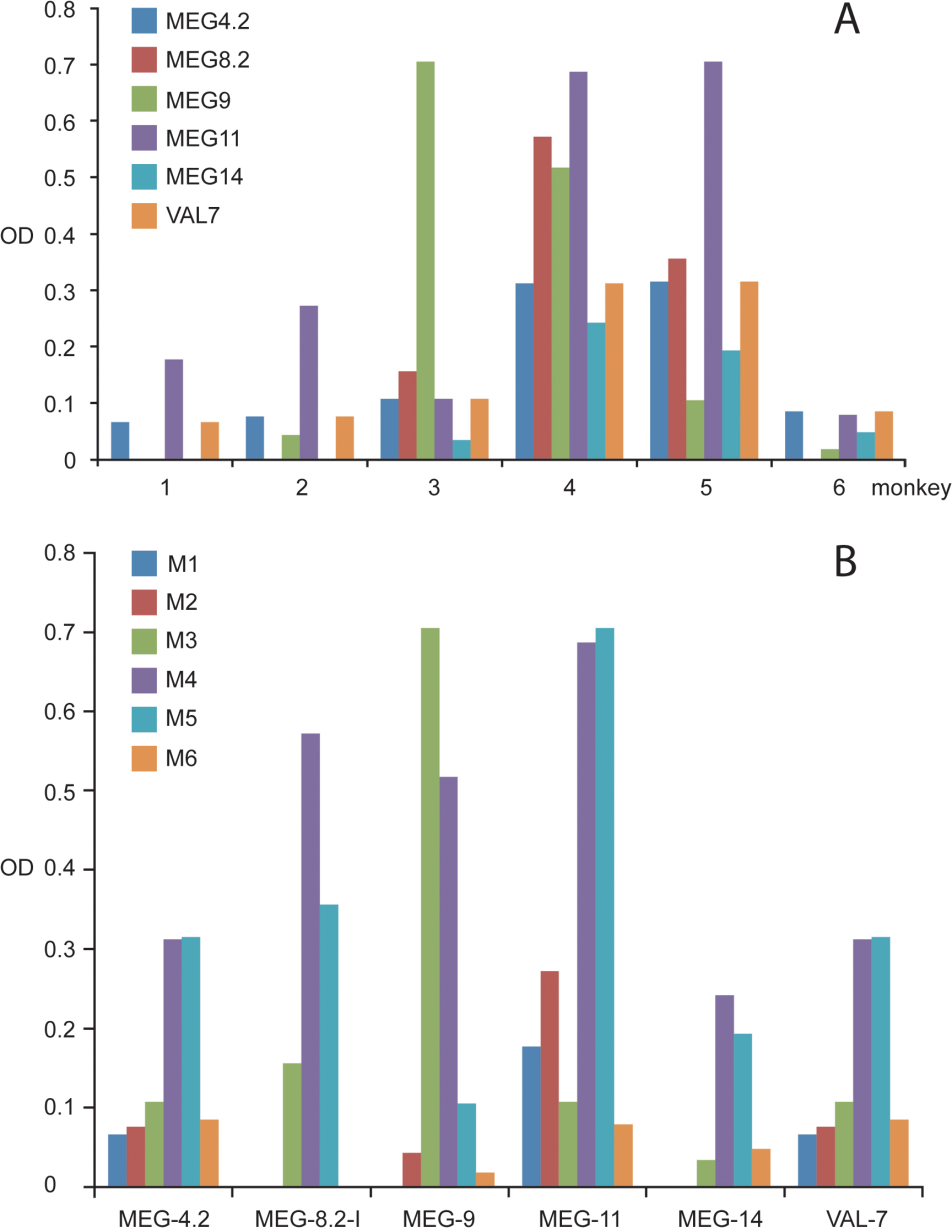
The monkey IgG response to esophageal gland proteins is heterogeneous. Data plotted by (A) animal and (B) proteins, respectively. (A) shows that all monkeys responded to at least three esophageal proteins but variation was apparent between individuals. Monkeys 4 and 5 recognized all tested proteins, some with a strong response while the repertoire of Monkeys 1, 2 and 6 was much less impressive. (B) shows that three esophageal proteins, MEG-4.2, MEG-11and VAL-7 were recognized by all monkeys, although with different intensities. MEG-9 was recognized by IgG from five monkeys, MEG-14 by four and MEG-8.2 by three.

We also investigated the IgG response of individual rhesus macaques to the demonstrated esophageal gland secreted proteins. For this purpose, ELISA plates were coated with the same synthetic peptides/recombinant proteins used to elicit specific antibodies in rats for immunocytochemistry. The resulting data were displayed by monkey number (Fig 9A) and by synthetic peptides or recombinant constructs (Fig 9B) on the x axis. All monkeys made a detectable response to one or more targets but there was considerable variation among individuals. All six monkeys made a response to MEG-4.2, MEG-11 and VAL-7, five to MEG-9, four to MEG-14 and three to MEG-8.2. No monkey responded to the short MEG-4.1 peptide. The maximum intensity of response to a given construct varied greatly among animals. Data plotted by monkey revealed the overall response to esophageal proteins in descending order, 4, 5, 3, 6, 2, 1 but the impressive repertoire of monkeys 4 and 5 did not correlate with worm burdens at perfusion, these being high and low burden, respectively.

## Discussion

Initially, the rhesus macaque behaves as a permissive host for *S*. *japonicum*, with approximately 75% of applied cercariae being recovered as mature adults six weeks post infection, worm bodies of both genders being even longer than those from the permissive rabbit host [7]. Viable eggs are laid in large numbers and granulomatous liver pathology ensues [7]. However, unlike most other hosts of this zoonotic parasite, the monkeys develop a self-cure response, so that by 20 weeks or so only a small fraction of the original worm population remains [7]. In our study, the data on faecal egg output showed that a robust infection was established in all animals after cercarial exposure. The CAA level also provided good evidence for establishment of a considerable worm population. The body weight reductions that followed oviposition plus the diarrhea and inappetence at week 8 (typical clinical symptoms of acute schistosomiasis) were independent evidence for the magnitude of infection, whilst the subsequent increase in weight revealed that by week 12 the impact of the infection was waning. The coincident reduced egg output while CAA levels remained high, showed that worms were still present but already manifesting impaired reproductive capacity. Indeed, females recovered from rhesus macaques and permissive hosts clearly fell into two distinct populations, the former being shorter and having fewer eggs, whilst the latter were longer with more eggs.

The much smaller size of females at week 22 compared to their counterparts at week 6 [5] and their declining reproductive capacity is striking; it is clear that their bodies shrink as their feeding is impaired. Nevertheless, the tegument and gastrodermis were intact with negligible changes in structure observed. The fact that the less pigment a female had in its gut the shorter it was, again demonstrates that blood feeding is vital for worm health, particularly in females. Even the rhesus females that we classified as ‘black’ actually contained much less hemozoin pigment than mature females from rabbit and mouse hosts, indicating that even the most active were processing less blood than normal worms. Furthermore, the lower female:male sex ratio suggests that females are more susceptible to rhesus macaque responses. The impact of rhesus host responses on males appears less marked. On average they were only 30% shorter than normal males, while many could still produce sperm and inseminate females, even debilitated females. These gender discrepancies can be explained by differences in the relative dependence of males and females on feeding via the tegument versus the gut [21]. The male worm relies on massive trans-tegumental uptake of glucose to supply its energy requirements (4.5 x its own dry weight per day), with correspondingly less reliance on blood ingestion via the gut. Conversely, the female ingests four times her own dry weight in blood protein via the gut, with relatively much lower glucose uptake across the tegument. On this basis females would be more susceptible than males to immune effector mechanisms operating against esophageal functions that disrupted feeding. Whatever the mechanism, the upshot appears to be that males survive in rhesus macaques better than females; however, both sexes eventually succumb.

The marked changes recorded in the reproductive system of females are most likely attributable to reduced nutrient uptake (rather than the converse). The only other major changes in cellular morphology, detected by electron microscopy, were in the esophageal region. Whilst not ruling out other causes for worm demise, the most economical explanation is that rhesus antibodies target the still poorly understood esophageal functions. Indeed, the anterior esophageal gland was only formally described in late 2014 [18]. Progress in understanding how neutralizing antibodies might block the functions of esophageal proteins is hampered by the lack of knowledge about the secretions and the roles of individual constituents in blood processing. Our observations on the structure of the anterior esophagus of rhesus worms reveal striking differences from the structures we have described in comparable worms from the permissive murine host [18]. There is a marked reduction in the surface area of the lining, with more fragile corrugations and fewer spaghetti filaments, which could reflect immunological pressure associated with the self-cure mechanism. However, the principal changes appear to be in the vesicle secretory process itself.

Major discrepancies in the morphology of worms from rhesus macaques compared with normal worms are the swollen expanded tips of the anterior corrugations packed with greatly enlarged light vesicles, and the deposition of material on the flattened plates partially or completely obscuring the surface pits. Our data strongly suggest that the deposits comprise host antibody and also fibrin. The pits on the flattened plates may be homologous to porosomes [32], where vesicle docking occurs on apical plasma membranes in many cell types [32,33]. This process involves formation of a fusion pore/channel [34,35] which can remain open for an extended period, permitting egress of contents. Furthermore, in some cell types, vesicles adjacent to the plasma membrane fuse with it while underlying vesicles fuse with their neighbors to form a chain of secretion through the single fusion pore [36]. In worms from rhesus macaques, it is plausible that antibodies against one or more vesicle constituents enter via the fusion pore to form immune complexes that block discharge into the esophageal lumen. Staining of the oval blobs in the esophagus lining indicates that this may indeed occur. Giant vesicle formation could then result from vesicle to vesicle fusion after discharge of contents is blocked. Identification of the proteins secreted from the newly designated anterior esophageal gland is now in progress.

Our TEM observations suggest that malfunction also occurs in the posterior esophageal compartment. One factor may be the close adherence of post esophageal plates, potentially cross-linked by antibodies to peptide or glycan epitopes. Indeed, the average 17 nm distance between the outer surfaces of adjacent plates approximates to the flexible distance between the two antigen binding sites on a single IgG molecule (~15 nm). Moreover, adherence of the plates would prevent the secretions of the posterior esophageal gland, such as the MEGs and VAL-7 from reaching the central lumen. A second factor is the plug of tethered leucocytes present in the posterior esophageal lumen. In worms from permissive hosts we have demonstrated by video microscopy that blood is able to flow round this plug unhindered [17,21]. Our observation of a more substantial plug in some rhesus worms suggests a vital process has been inhibited such that build-up of tissue debris occurs. Undoubtedly, such inhibition will interfere with blood processing in the posterior esophagus.

It has been documented that debility and elimination of adult worms does not occur in monkeys with a light infection, where egg output remains unchanged over long periods [9,37]. This is one of the strongest arguments for an immunological basis to self-cure but correlative measurements of antibody level were not made in these early studies. Based on our premise that antibody is responsible for worm elimination, we investigated the nature of the antigenic targets. Confocal microscopy on permeabilized worms revealed the tegument, esophagus and gut were all prominent sites of IgG binding in live worms recovered from rhesus macaques. Note that antibody does not bind to internal (i.e. intracellular) proteins in these intact worms in vivo. In the context of other potential targets we have evaluated the antibody responses to a protein array comprising 172 putative tegument constituents [38]. A significant response was detected to eight proteins, only one of which (SGTP1) is a verified constituent of the apical plasma membrane in *S*. *mansoni*. In the current study we elected to focus on the esophagus because of its newly appreciated role as the site of secretions that mediate the initial stage of blood processing [17]. In addition, its structure was modified in rhesus worms that showed a very obvious reduction in blood feeding. Probing a conventional SWAP preparation with the rhesus serum provided a general profile of the host response to soluble somatic antigen preparations comprising highly immunogenic, and internal proteins [39]. When we generated a protein fraction from *S*. *japonicum* heads, estimated to give a 50-fold enrichment of esophageal gland proteins, the principal constituents were still of cytosolic and cytoskeletal origin so we were compelled to take a piecemeal approach to investigate the reactivity of the esophageal proteins we had identified.

We previously showed that four genes (SmMEG-4.1, SmMEG-4.2, SmMEG-14, SmVAL-7) encoding secreted proteins were specifically expressed in the *S*. *mansoni* esophageal gland, and that SjMEG-4.1 protein was present in the posterior esophageal gland of *S*. *japonicum* [17]. In the current study we used immunocytochemistry to demonstrate that three more homologues of the *S*. *mansoni* genes (SjMEG-4.2, SjMEG-14 and SjVAL-7) plus three novel SjMEGs 8.2, 9 and 11, are localized in and secreted from the posterior gland of *S*. *japonicum*. (SjMEG-14 is predicted to be membrane-anchored.) The way in which the secreted MEG proteins we identified interact with components of ingested blood to initiate its processing, remains to be established. However, it is noteworthy that MEG-4.1 from *S*. *mansoni* was shown to heavily O-glycosylated [17] and both MEGs 4.1 and 8.2 from *S*. *japonicum* are predicted to have similar properties. Furthermore, the close association we observed between these two proteins and the plug of leucocytes in the posterior lumen suggests that their combined physical properties may be responsible for cell tethering.

Our ELISA data revealed that the IgG responses of the monkeys to the seven targets differed, perhaps unsurprising, as they are from an outbred population. Nevertheless, all monkeys reacted with the SjMEG-4.2 C-terminus, MEG-11 and VAL-7. No reaction to MEG-4.1 was detected in any monkey, probably due to the short peptide we used to coat the plates. It is worth noting that this approach is most likely to detect linear epitopes, whilst antibodies that bind in vivo to native proteins in live worms can potentially react with conformational, linear and glycan epitopes. The co-localisation approach does not discriminate between two or more superimposed secreted targets in the esophagus lumen, whereas detection of a free protein not coincident with the antibody staining pattern may rule it out as a mediator of the self-cure process. On this basis, MEG-4.2, which did overlay in some places, appears the least likely candidate while the remainder (MEGs 4.1, 8.2, 9, 11 and VAL-7) must be considered potential targets of the self-cure process. It is intriguing that IgG binding has also been detected in the esophageal lumen of worms from mice [18], yet they do not eliminate adult worms. We suggest this is due to the intensity and/or the specificity of the response and we are currently seeking differences in the reactivity of rhesus, mouse and rabbit infection sera to esophageal secretions.

Our results emphasize the central place of the worm esophagus in the mechanism of self-cure mounted against adult worms by the rhesus macaque. They provide a clear explanation for the cessation of feeding that leads to worm starvation and death and they implicate the protein products of MEGs as likely targets. A recent study has revealed that this group of genes has been subjected to greater selective pressure than any other during evolution of the Genus *Schistosoma* [40]. It is tempting to conclude that the host immune system has applied the pressure mainly on the esophagus, where the battle has been waged. Our immediate task is to identify more esophageal secreted proteins, especially from the anterior gland where the morphological changes are most visible.

Targeting esophageal proteins provides a novel, hitherto unexplored, route to an effective schistosome vaccine. Furthermore, indications from early studies in the rhesus macaque are that once the immunity has been induced, it is long lasting [13]. It is also possible that when subsequent invasions of worms reach the blood-feeding stage in the portal tract they have the capacity to boost the established protective response. This is not a feature found in the immunity to *S*. *mansoni* induced in another primate host, the baboon, by radiation-attenuated cercariae where the protection rapidly wanes and is not boosted by challenge larvae [41]. There are pointers from the literature that self-cure occurs in other natural hosts of *S*. *japonicum*, including water buffalo [42], but on a longer time scale, although it is not clear whether this has an immunological basis. Analysis of host antibody responses to a broad spectrum of esophageal secreted proteins should therefore provide the key to select the best vaccine candidates. In this context our findings on the immunological-based self-cure mechanisms in rhesus macaques and their association with esophageal secretions should open a new chapter for vaccine development against this important helminth zoonosis.

## Supporting Information

S1 Fig Worm burden at perfusion and a comparison of the morphology of worms from two hosts. (A) The worm burden of each monkey at perfusion, the females classified visually by the amount of gut pigment. Note that although some worms were still paired, the female partner may be anaemic. A wide variation is evident in the number of worms recovered from the monkeys at perfusion, #s 1 and 4 having a burden of more than 100, whilst #s 6 and 2 had only 15 and 16 worms, respectively. (B) Worms recovered from rhesus macaques at 22 weeks differed significantly in appearance from normal mature worms recovered from rabbits at 6 weeks (C). Females from rabbits (C, arrowed) were uniformly black and healthy, whilst those from rhesus macaques (B, arrowed) were pale and shrunken. Scale bars: 5 mm (B, C).
(TIF)

S2 Fig Detailed alterations in the morphology of the female reproductive system revealed by confocal microscopy. Confocal images of oviduct and vitelline duct in females from rhesus macaques (A, B and C) and mouse (D). Both White group (A) and Black group (B) show massive numbers of sperm adherent to a region of oviduct (star adjacent), vitelline cells (arrowed) in the vitelline duct being smaller and spaced apart. (C) shows the oviduct (OD) of a White group female is enlarged greatly. (D) shows that oviduct and vitelline duct of a female from permissive host are usually well hidden in deep tissues; bigger cells (arrowed) are present in the vitelline duct and sperm (star adjacent) are very localised in the oviduct. Scale bars: 20 μm (A, B, C, D).
(TIF)

S3 Fig Morphology of the anterior esophagus in adult *S*. *japonicum* from permissive hosts. Figures reproduced with accompanying legends from Li et al., 2014, Parasites & Vectors 7, 565. Part 1, Confocal and SEM images. Part 2, TEM images.
(TIF)

S4 Fig Fibrin colocalised with host IgG in the deposits on the anterior esophageal lining. Sections of worm heads from rhesus macaques stained by FITC labeled anti-fibrin antibody and Cy3 labelled anti-rhesus IgG antibody were counterstained with DAPI (blue) to highlight the nuclei. The triplet images display the distribution of fibrin staining (green, A), the rhesus IgG (red, B), and the two overlaid in the third (C). The lemon yellow color indicated areas of colocalization (C), free green staining was also evident indicating not all fibrin was superimposed by host antibody. Scale bar: 20 μm (A, B, C).
(TIF)

S5 Fig Cellular morphology characteristic of the post esophageal lumen of worms from permissive hosts. A Confocal image of a rabbit worm head section stained with DAPI and phalloidin to illustrate nuclei and muscle, showing a cell plug (starred) in the center of the posterior esophageal lumen (PEL). AEL, anterior esophageal lumen; TG, transverse gut. B, TEM image of a mouse worm shows the posterior esophageal lumen (PEL) was clear with visible debris (D) and the plates (P) were well spaced apart. Scale bar: 20 μm (A), 2 μm (B).
(TIF)

S6 Fig Validation of purified recombinant proteins SjMEG-8.2 and SjVAL-7. A. Purification of recombinant proteins revealed by SDS-PAGE: (a) SjMEG-8.2 fused with Im-9 partner (combined MW 20.3kDa) to improve expression and solubility; (b) SjVAL-7 solubilized in urea (MW 22kDa). B. Tandem mass spectrometry of gel bands to confirm identity of expressed proteins.
(TIF)

S1 Table Estimation of mature worm burden from peak egg output. (XLSX)

S2 Table Amino acid sequences of synthetic peptides and their GenBank accession number. (XLSX)

S3 Table Soluble protein composition of a worm head preparation. (XLSX)
