# Supplementary figures and images for: Evidence That Rhesus Macaques Self-Cure from a Schistosoma japonicum Infection by Disrupting Worm Esophageal Function: A New Route to an Effective Vaccine?
Source: PLoS Negl Trop Dis. 2015 Jul 10;9(7):e0003925. doi: 10.1371/journal.pntd.0003925 (PMC4498593; doi:10.1371/journal.pntd.0003925)

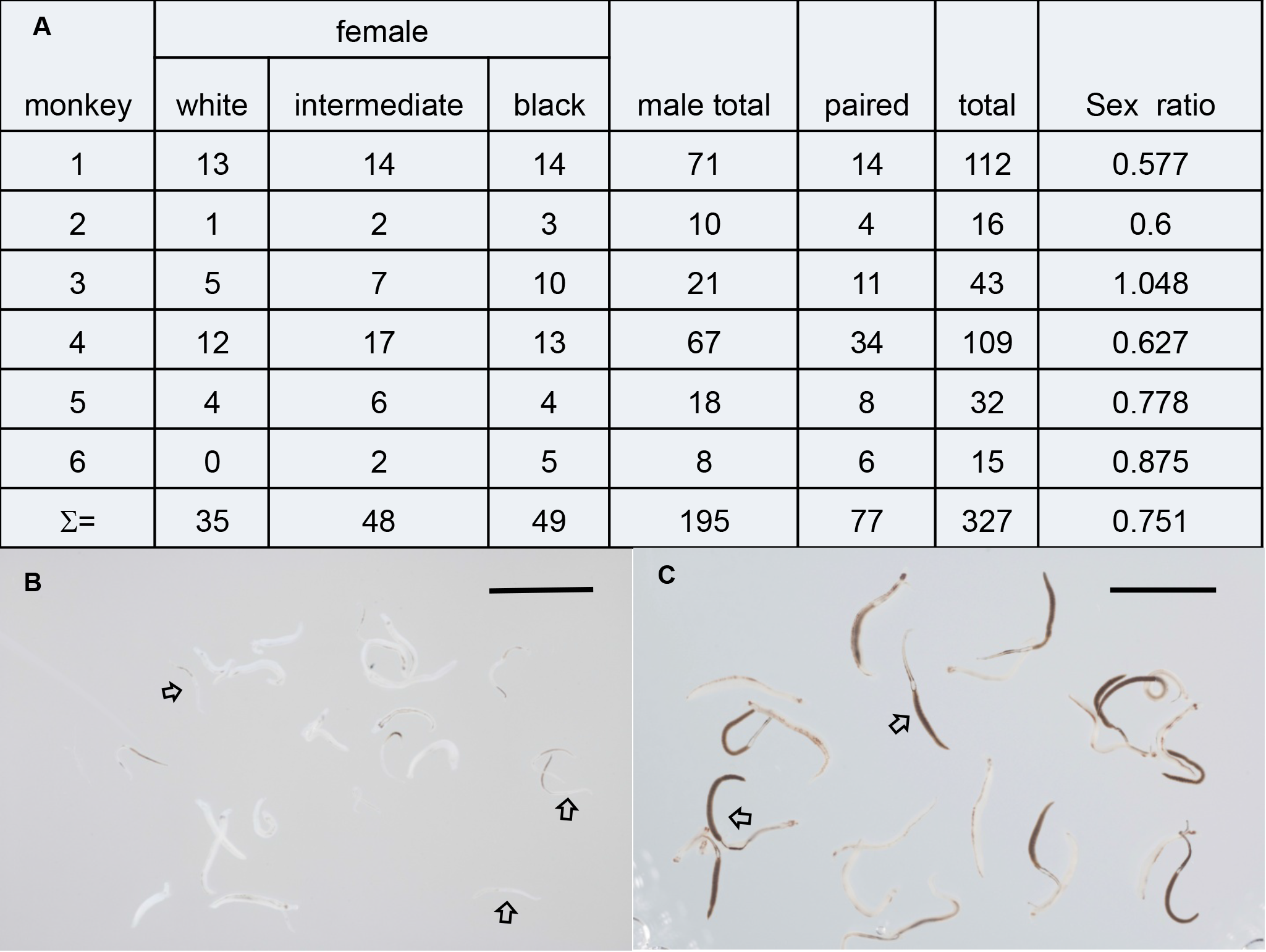

Supplement: S1 Fig — (A) The worm burden of each monkey at perfusion, the females classified visually by the amount of gut pigment. Note that although some worms were still paired, the female partner may be anaemic. A wide variation is evident in the number of worms recovered from the monkeys at perfusion, #s 1 and 4 having a burden of more than 100, whilst #s 6 and 2 had only 15 and 16 worms, respectively. (B) Worms recovered from rhesus macaques at 22 weeks differed significantly in appearance from normal mature worms recovered from rabbits at 6 weeks (C). Females from rabbits (C, arrowed) were uniformly black and healthy, whilst those from rhesus macaques (B, arrowed) were pale and shrunken. Scale bars: 5 mm (B, C). (TIF) [file pntd.0003925.s001.tif]

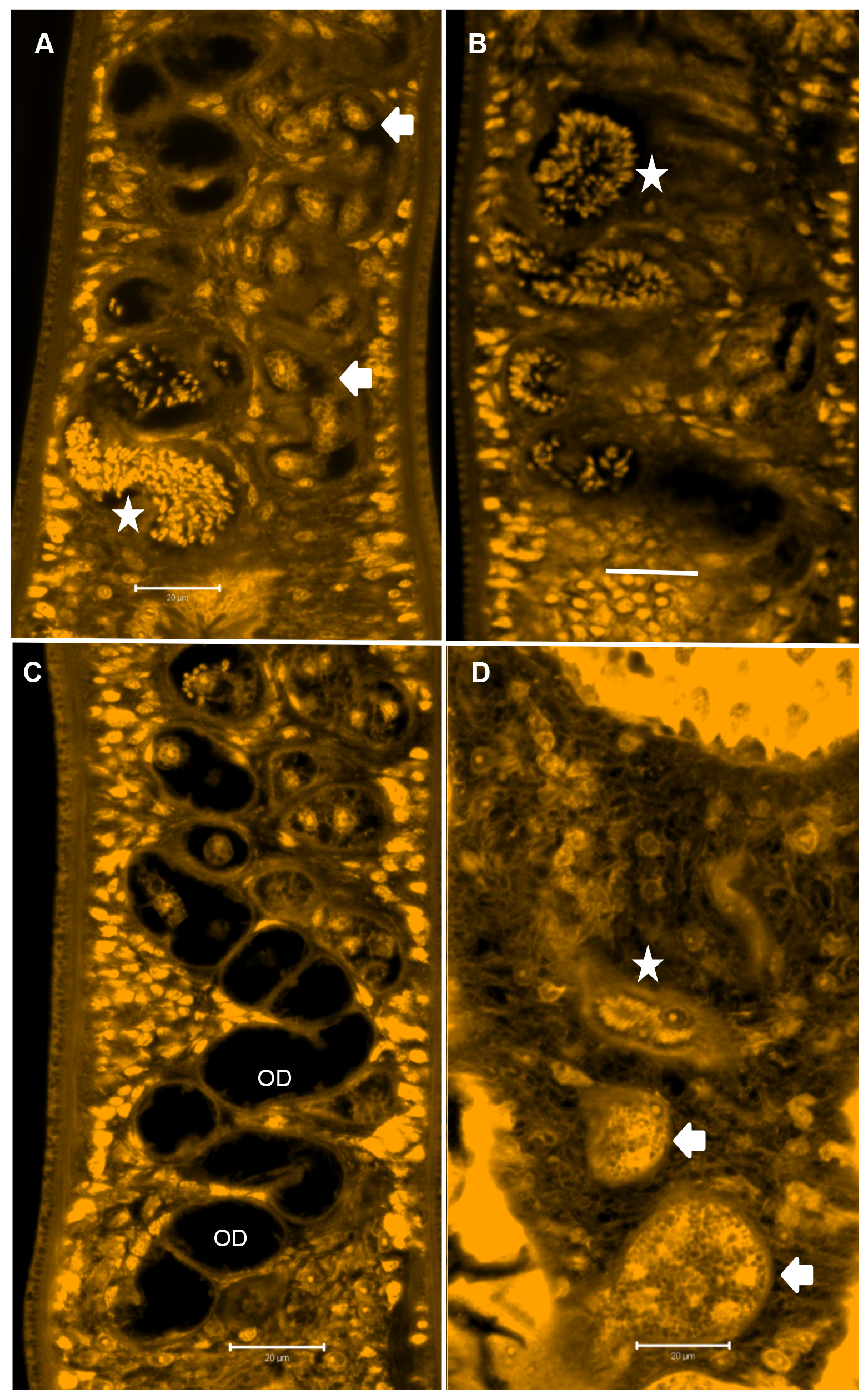

Supplement: S2 Fig — Confocal images of oviduct and vitelline duct in females from rhesus macaques (A, B and C) and mouse (D). Both White group (A) and Black group (B) show massive numbers of sperm adherent to a region of oviduct (star adjacent), vitelline cells (arrowed) in the vitelline duct being smaller and spaced apart. (C) shows the oviduct (OD) of a White group female is enlarged greatly. (D) shows that oviduct and vitelline duct of a female from permissive host are usually well hidden in deep tissues; bigger cells (arrowed) are present in the vitelline duct and sperm (star adjacent) are very localised in the oviduct. Scale bars: 20 μm (A, B, C, D). (TIF) [file pntd.0003925.s002.tif]

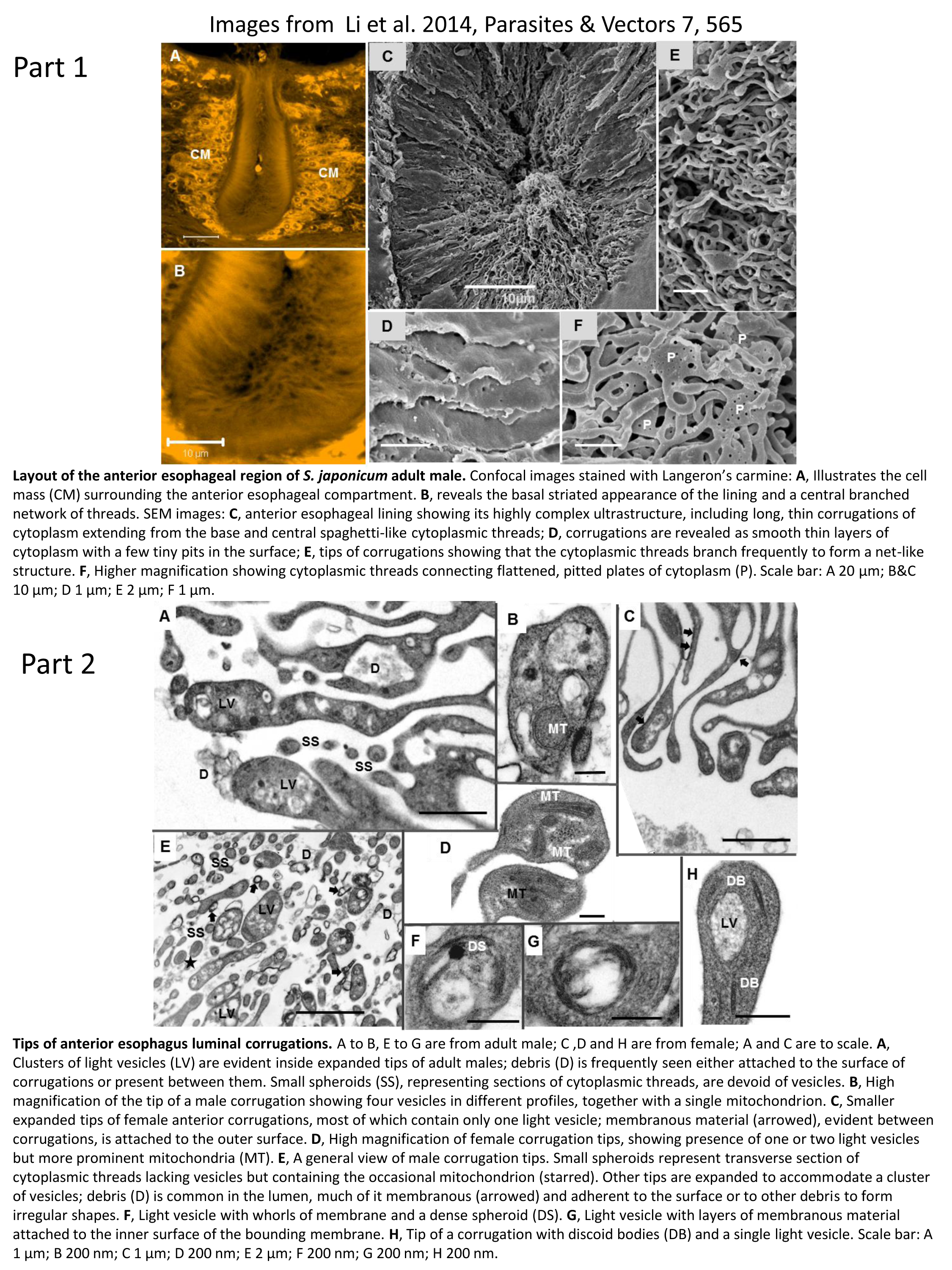

Supplement: S3 Fig — Figures reproduced with accompanying legends from Li et al., 2014, Parasites & Vectors 7, 565. Part 1, Confocal and SEM images. Part 2, TEM images. (TIF) [file pntd.0003925.s003.tif]

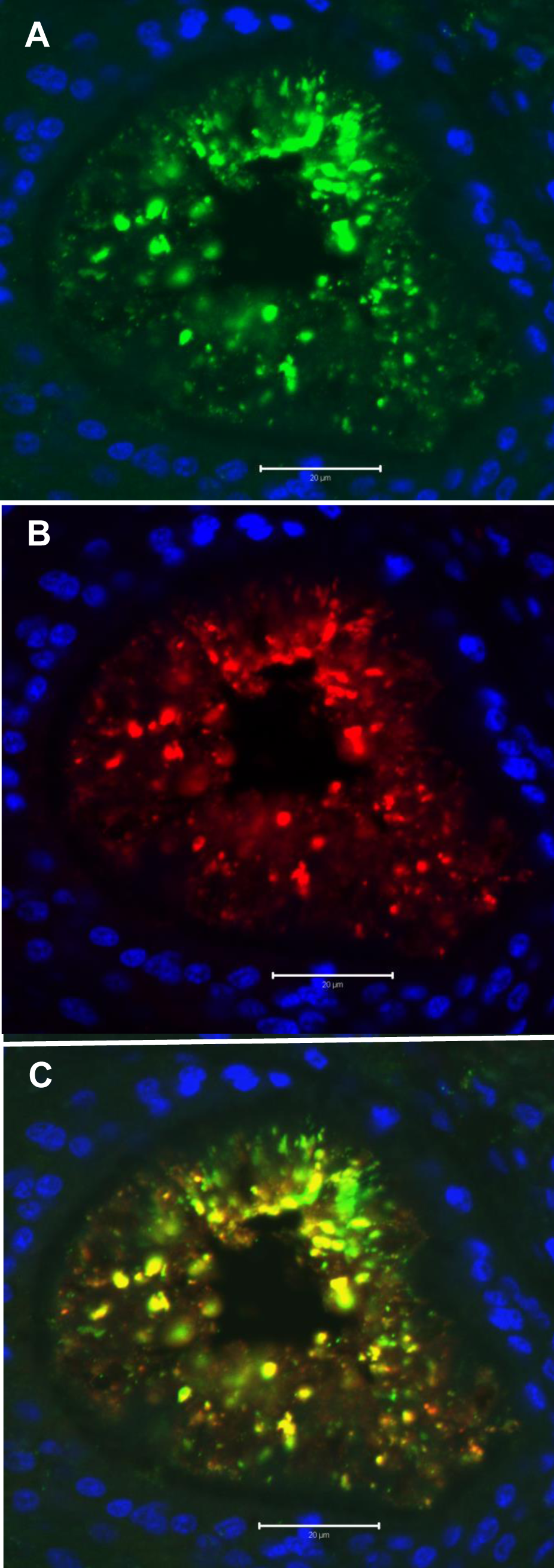

Supplement: S4 Fig — Sections of worm heads from rhesus macaques stained by FITC labeled anti-fibrin antibody and Cy3 labelled anti-rhesus IgG antibody were counterstained with DAPI (blue) to highlight the nuclei. The triplet images display the distribution of fibrin staining (green, A), the rhesus IgG (red, B), and the two overlaid in the third (C). The lemon yellow color indicated areas of colocalization (C), free green staining was also evident indicating not all fibrin was superimposed by host antibody. Scale bar: 20 μm (A, B, C). (TIF) [file pntd.0003925.s004.tif]

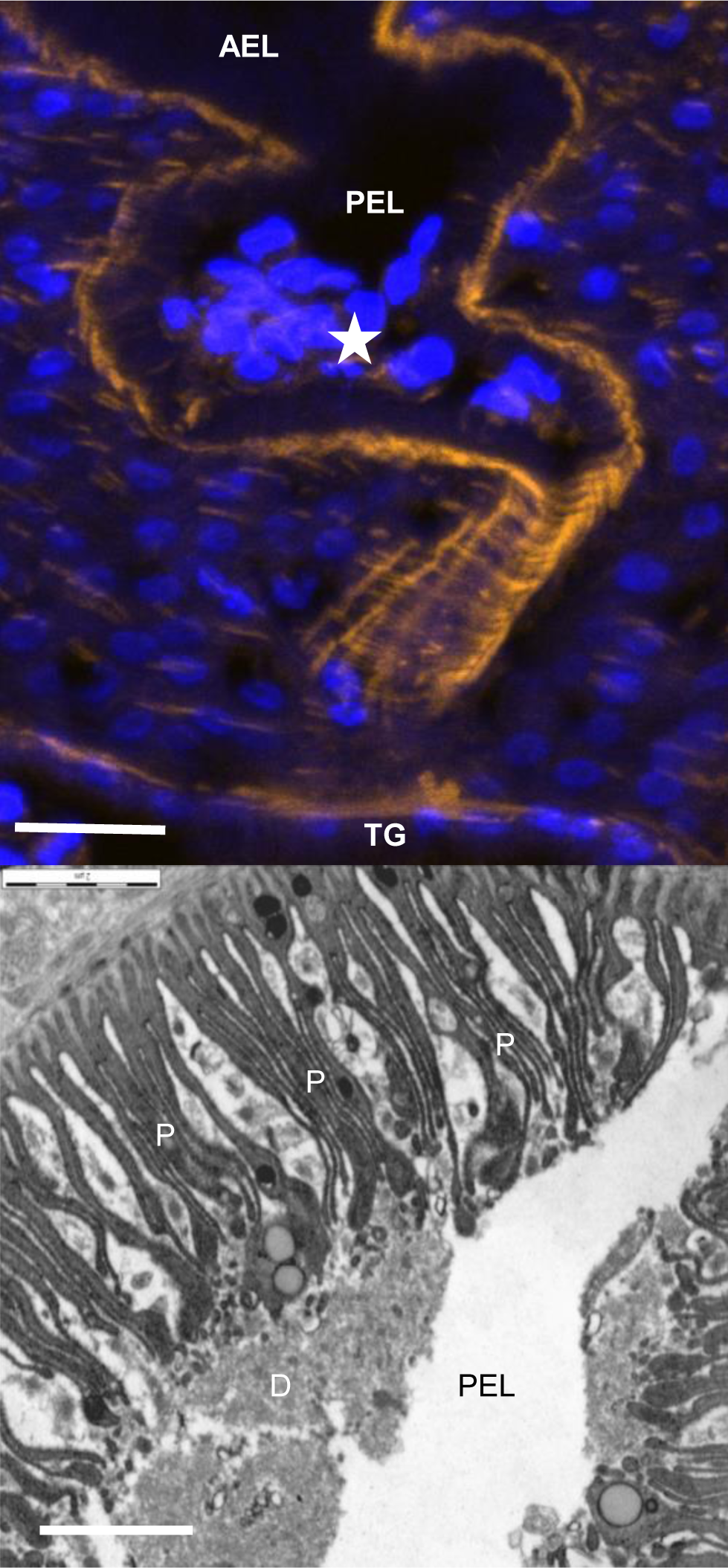

Supplement: S5 Fig — A Confocal image of a rabbit worm head section stained with DAPI and phalloidin to illustrate nuclei and muscle, showing a cell plug (starred) in the center of the posterior esophageal lumen (PEL). AEL, anterior esophageal lumen; TG, transverse gut. B, TEM image of a mouse worm shows the posterior esophageal lumen (PEL) was clear with visible debris (D) and the plates (P) were well spaced apart. Scale bar: 20 μm (A), 2 μm (B). (TIF) [file pntd.0003925.s005.tif]

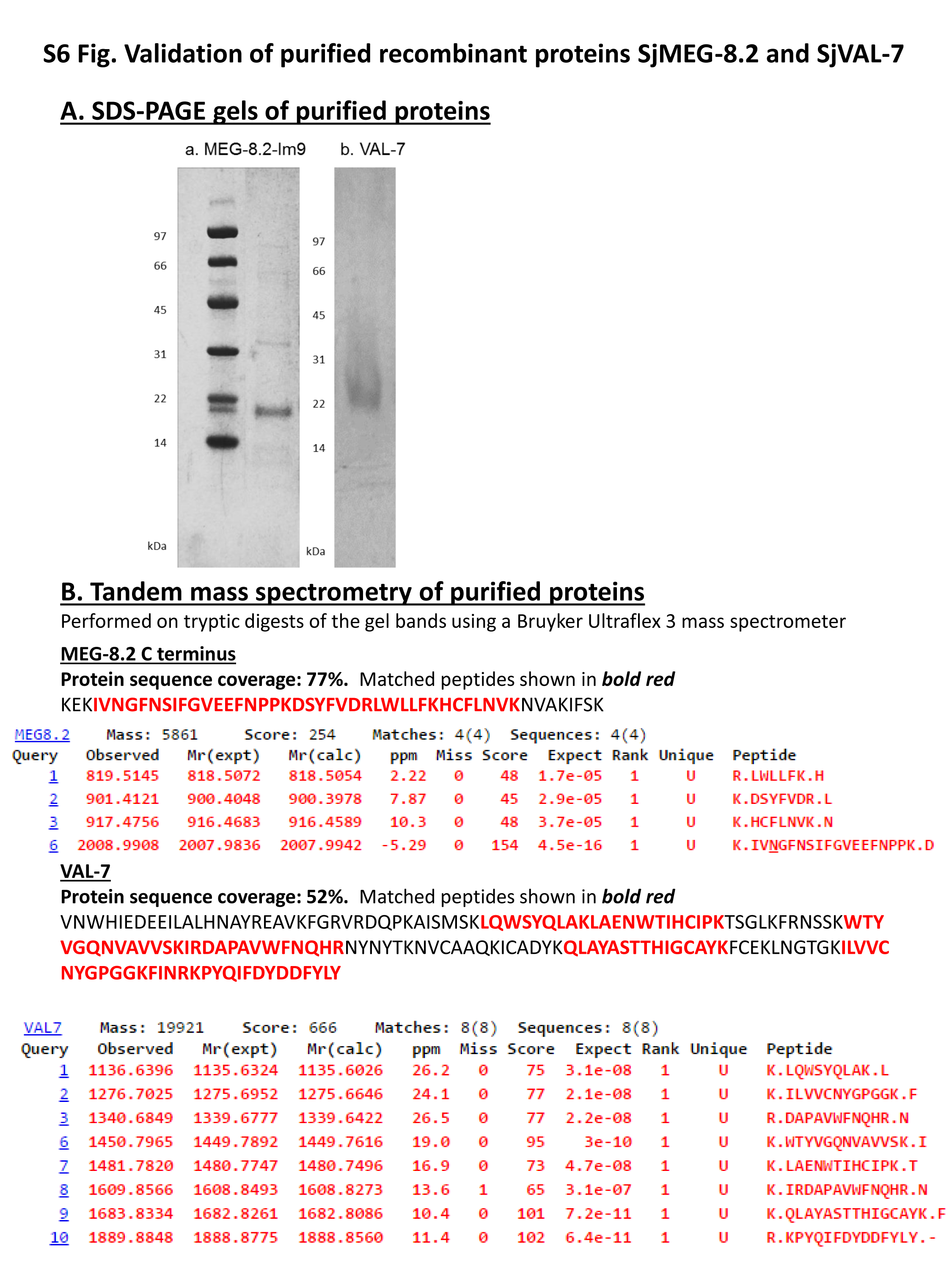

Supplement: S6 Fig — A. Purification of recombinant proteins revealed by SDS-PAGE: (a) SjMEG-8.2 fused with Im-9 partner (combined MW 20.3kDa) to improve expression and solubility; (b) SjVAL-7 solubilized in urea (MW 22kDa). B. Tandem mass spectrometry of gel bands to confirm identity of expressed proteins. (TIF) [file pntd.0003925.s006.tif]
